# Carrier‐Free Nanocapsule with Dual‐Target Capacity for Synergistically Restoring Inflammatory Microenvironment and Microbiota Dysbiosis in Colitis

**DOI:** 10.1002/advs.202500001

**Published:** 2025-07-12

**Authors:** Yingjie Chen, Yuhan Gao, Kaiyuan Huo, Zhaofan Jin, Wenchao Wang, Xingjie Zan, Yanlong Liu, Limeng Zhu, Jianfeng Yang

**Affiliations:** ^1^ Cixi Biomedical Research Institute Wenzhou Medical University Ningbo Zhejiang 315300 China; ^2^ Wenzhou Institute University of Chinese Academy of Sciences Wenzhou Zhejiang 325001 China; ^3^ Postgraduate training base Alliance of Wenzhou Medical University Wenzhou Zhejiang 325000 China; ^4^ School of Pharmacy Wenzhou Medical University Wenzhou Zhejiang 325035 China; ^5^ School of Mental Health Wenzhou Medical University Wenzhou Zhejiang 325000 China; ^6^ Department of Gastroenterology, Affiliated Hangzhou First People's Hospital School of Medicine, Westlake University Hangzhou Zhejiang 310000 China

**Keywords:** Dual‐targeting strategy, Gut homeostasis, Inflammatory bowel disease, ROS scavenging

## Abstract

Inflammatory bowel disease (IBD) is a chronic inflammatory disease with limited therapeutic outcomes. Macrophages are the key gatekeepers of intestinal immune homeostasis and have vital influence on IBD. Hence, macrophages are recognized as attractive targets to develop new therapeutic. However, the therapy development has proven challenging due to the malignant biological chain between macrophage immune hyperresponsiveness and dysbiosis of intestinal microflora. Herein, a carrier‐free nano‐drug, PCNPs@PEG‐Man, with dual‐targeting function, is produced due to IBD lesion‐specific positive charge and high expression of mannose receptor. With super resistance against extreme intraluminal conditions, PCNPs@PEG‐Man showes stable ROS‐scavenging properties thereby. Notably, the dual‐targeting strategy enhances the endocytosis efficiency and intestinal retention time of the drug, which is conducive to the downregulation of pro‐inflammatory factors, upregulation of anti‐inflammatory factors, and repair of the intestinal barrier. Additionally, it reshaped the dysbiosis of intestinal bacteria, revealing an optimized gut flora composition of probiotics. The mechanism of the carrier‐free nano‐drug mainly involves the elimination of oxidative stress, promoting macrophage M2 polarization, and restoring gut homeostasis. The synergistic effect inherent in this dual‐targeting system presents an effective and safe approach to managing IBD, providing new insights into the treatment of intestinal ROS‐mediated diseases associated with microbiota dysbiosis.

## Graphical abstract

This manuscript presents a carrier‐free nanomedicine PCNPs@PEG‐Man with dual‐targeting for IBD. It resists harsh intestinal conditions, reduces oxidative stress, enhances drug uptake and retention, modulates inflammation, and reshapes gut microbiota, offering a safe and effective IBD therapy.

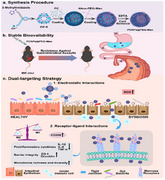

## 1 Introduction

Inflammatory bowel disease (IBD) encompasses a spectrum of challenging‐to‐manage disorders marked by persistent and recurrent intestinal inflammation, including ulcerative colitis and crohn's disease.^[^ ^1^ ^]^ In North America and Europe, over 1.5 million and 2 million people, respectively, have been diagnosed with IBD since the turn of the century, with incidence rates rapidly increasing in Asia, particularly in countries such as China and India.^[^ ^2^ ^]^ IBD not only significantly affects patients' quality of life but can also lead to severe complications such as intestinal perforation and cancer.^[^ ^3^ ^]^ Currently, pharmacological treatment options for IBD include anti‐inflammatory agents (e.g., 5‐ASA), immunosuppressive drugs (e.g., methotrexate), and biological therapies (e.g., infliximab).^[^ ^4^ ^]^ Nevertheless, these therapies are constrained by several limitations, including narrow targeting, a lack of specificity, the potential for liver and kidney toxicity at high doses, and the development of resistance and immunosuppressive effects with prolonged use.^[^ ^5^ ^]^ Consequently, there is a pressing need for the development of more effective strategies for IBD management.

Macrophages play a crucial role in maintaining intestinal immune homeostasis and preventing excessive immune responses during IBD.^[^ ^6^ ^]^ However, abnormal macrophage activation leads to overproduction of reactive oxygen species (ROS) at the lesion site, damaging proteins, lipids, and DNA, and compromising the intestinal barrier.^[^ ^7^ ^]^ This results in increased apoptosis and dysbiosis, further disrupting the gut microbiome's delicate balance.^[^ ^8^ ^]^ Activated macrophages perpetuate the inflammatory cycle by releasing pro‐inflammatory mediators such as IL‐1β, IL‐6, and TNF‐α, thus amplifying chronic inflammation.^[^ ^9^ ^]^ This reprogramming may reduce ROS levels, protect the intestinal barrier, alleviate tissue damage, and restore gut homeostasis.^[^ ^10^ ^]^

Natural products with antioxidant properties, such as polyphenols, have shown potential in treating inflammatory conditions like IBD.^[^ ^11^ ^]^ However, the oral administration of these compounds poses challenges related to their bioavailability and therapeutic effectiveness. Moreover, the controlled release and targeting characteristics of many current delivery systems are inadequate.^[^ ^12^ ^]^ Targeted drug delivery has emerged as a crucial area of research, as it has the potential to enhance specificity, efficacy, and therapeutic outcomes by concentrating drugs at disease sites while minimizing off‐target effects.^[^ ^13^ ^]^

Current strategies for targeting IBD treatment can be classified into two categories: passive targeting and active targeting.^[^ ^14^ ^]^ Passive targeting relies on physical interactions between the drug or carrier and the inflamed intestinal microenvironment to promote drug accumulation at sites of inflammation. For example, inflamed intestinal epithelial cells release positively charged proteins, creating an environment for negatively charged drugs or carriers to accumulate via electrostatic interaction.^[^ ^15^ ^]^ For instance, during the inflammatory, intestinal epithelial cells release positively charged proteins, thereby creating a potential for negatively charged drugs or carriers to accumulate at the site of IBD through an electrostatic interaction.^[^ ^16^ ^]^ However, the effectiveness of passive targeting is closely tied to the state of inflammation, and as inflammation subsides, its efficacy diminishes.^[^ ^1^, ^17^ ^]^ Conversely, active targeting employs specific interactions between a drug and overexpressed receptors on target cells, leading to more precise delivery. This approach typically utilizes pharmaceutical agents or carriers that bind to receptors, such as CD44 or CD206, initiating receptor‐mediated endocytosis to enhance delivery accuracy and efficacy.^[^ ^18^ ^]^ Unfortunately, active targeting may be hampered by disease heterogeneity and individual variability, potentially resulting in off‐target effects and reduced efficacy in patients lacking the necessary receptors.^[^ ^19^ ^]^

**Scheme 1:**
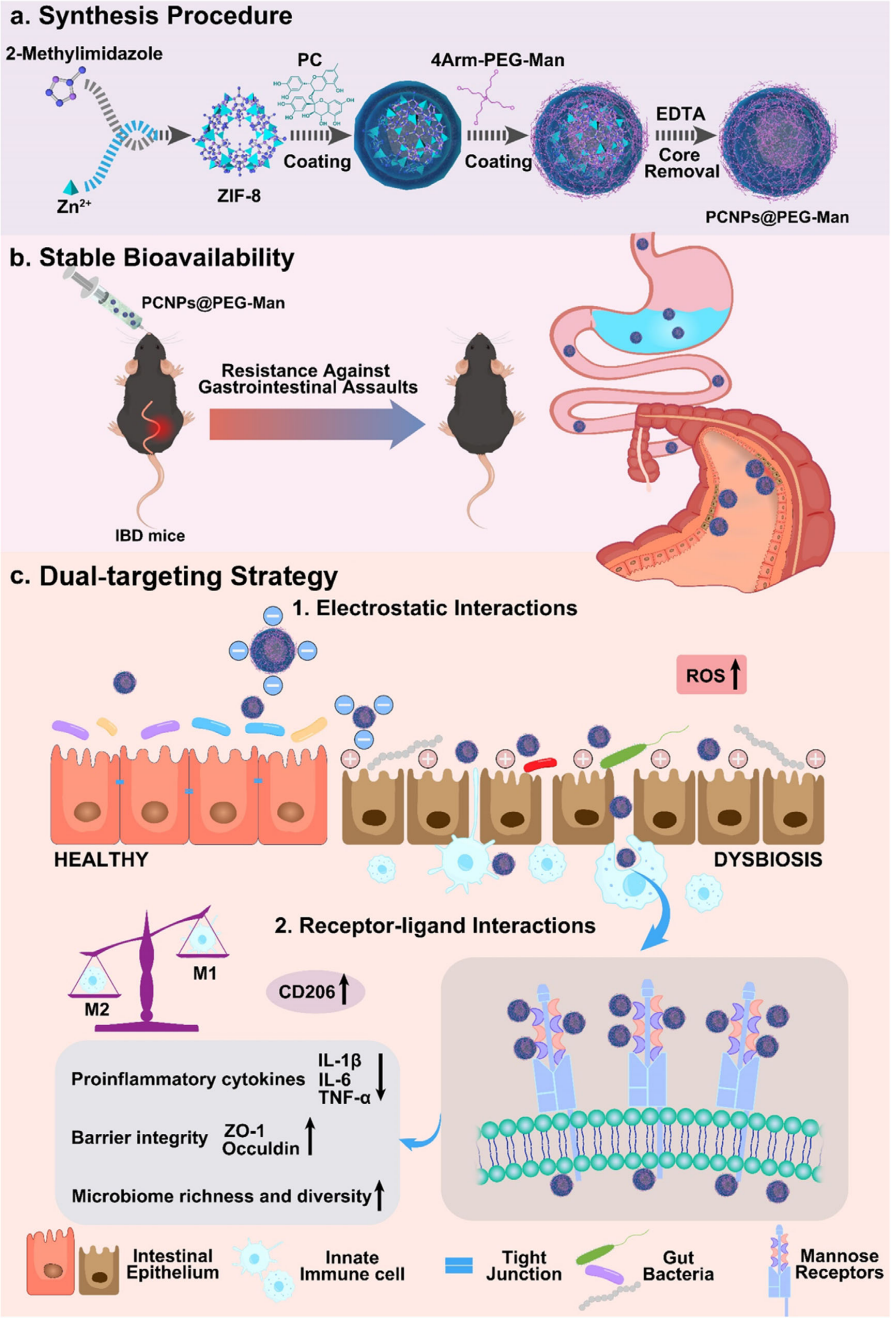
Schematic illustration of PCNPs@PEG‐Man for IBD treatment. a) Synthesis Procedure of PCNPs@PEG‐Man. b) Good gastrointestinal stability of Oral PCNPs@PEG‐Man. c) Dual‐targeting strategy of PCNPs@PEG‐Man and the therapeutic effect of IBD.

To address the above issues, herein, we presented a dual‐targeting system that modulates immune responses and maintains gut microbiota homeostasis for IBD treatment. The core material, proanthocyanidins, is a plant‐derived polyphenol known for its potent anti‐inflammatory, antioxidant, and immunomodulatory effects.^[^ ^20^ ^]^ However, as a monomer, its poor cellular internalization and susceptibility to degradation in vivo significantly reduce its bioavailability and therapeutic potential. Proanthocyanidin was formulated into a carrier‐free nano‐drug (PCNPs) using ZIF‐8 as a sacrificial template and modified with mannose receptor ligands (4Arm‐PEG‐Man), forming PCNPs@PEG‐Man (**Scheme** **1**a). The data presented herein demonstrated several advantages of PCNPs@PEG‐Man: 1) Enhanced disease‐site targetability under the IBD pathological microenvironment; 2) Reprogramming of macrophages by promoting M2 polarization, suppressing the release of inflammatory cytokines, and eliminating oxidative stress; 3) Improved intestinal barrier integrity via upregulation of tight junction proteins (ZO‐1, Occludin); and 4) Remodeling of gut microbiota, enhancing microbial diversity, eliminating pathogenic bacteria, and providing prebiotics (Scheme 1b). This dual‐targeting system, designed to regulate macrophage functions and restore balance in gut microbial communities, exhibited robust synergistic therapeutic potential for IBD and holds promise for clinical applications (Scheme 1c).

## 2 Results and Discussion

### 2.1 Preparation and Characterization of PCNPs and PCNPs@PEG‐Man

**Figure 1:**
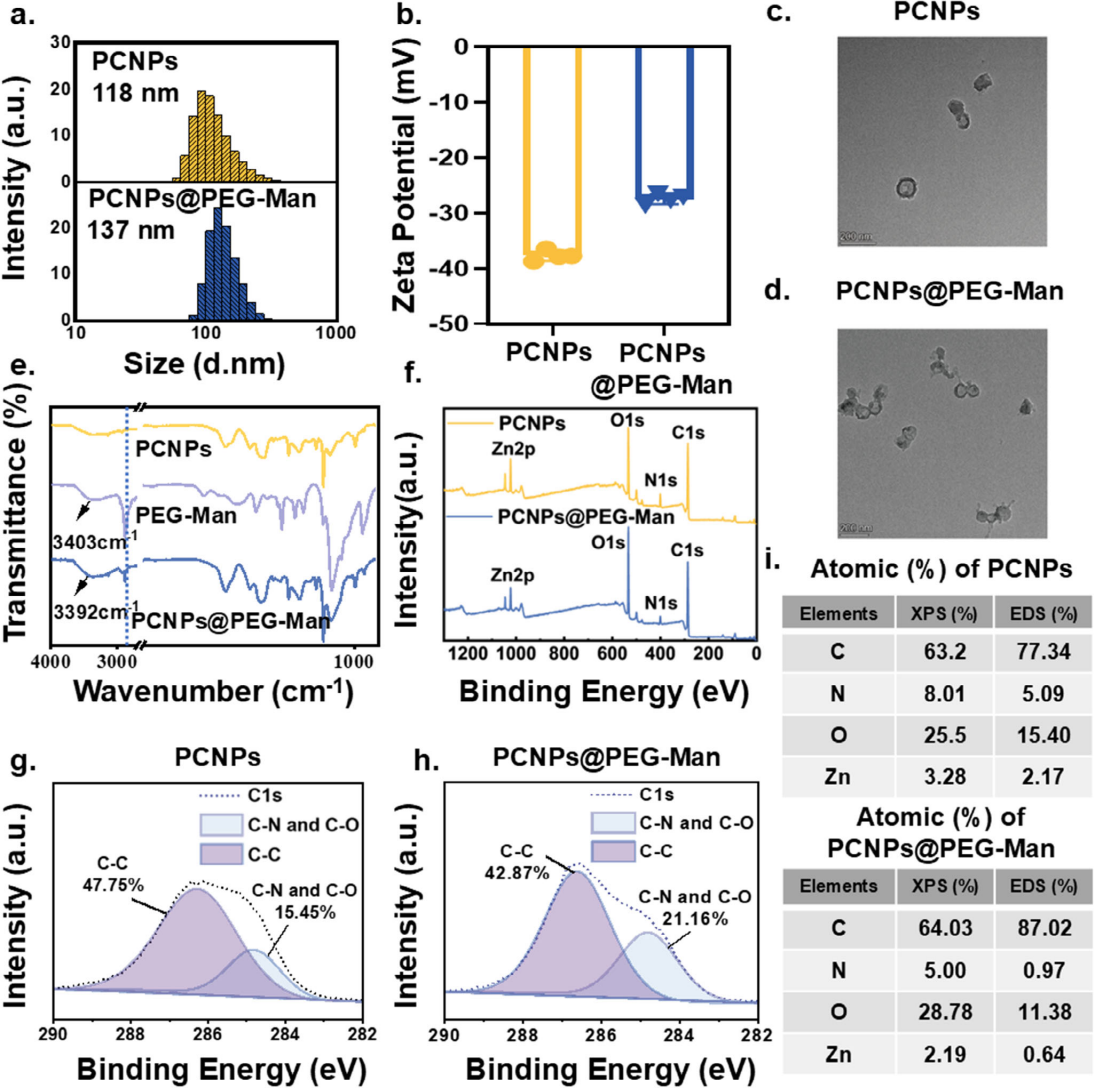
Characterization of PCNPs and PCNPs@PEG‐Man. a) Hydrodynamic diameter distribution by DLS, b) Zeta potential, c,d) representative TEM images, Scale bar: 200 nm, e) FTIR spectra, f) XPS spectra of PCNPs and PCNPs@PEG‐Man. Detailed XPS spectra of the C1s of g) PCNPs and h) PCNPs@PEG‐Man, for highlighting the change in the C1s core level after modification. i) The element composition ratio of XPS and EDS.

In this study, PCNP nanocapsules were synthesized through a multi‐step process. Subsequently, 4‐PEG‐Man (structural formula shown in Figure S1a, Supporting Information) was incorporated using a solvent stirring method.^[^ ^21^ ^]^ The detailed synthesis of PCNP and PCNPs@PEG‐Man nanocapsules is illustrated in Scheme 1a. Following the etching of the ZIF‐8 template, nanocapsules with a medium shell structure were formed. To start with, the hydrodynamic diameters of PCNPs and PCNPs@PEG‐Man nanocapsules were measured at 118 nm and 137 nm, respectively (**Figure** **1**a), indicating that PEG‐Man modification did not significantly alter the size. Zeta potential measurements showed values of ‐37.65 ± 0.82 mV for PCNPs and ‐27.48 ± 0.78 mV for PCNPs@PEG‐Man (Figure 1b), highlighting a substantial change in surface charge due to modification. Furthermore, the ultrastructure of the nanocapsules was examined using transmission electron microscopy. The result revealed that both PCNPs and PCNPs@PEG‐Man exhibited spherical and concave shapes (Figure 1c,d), underscoring their good dispersion and morphology. Elemental analysis through energy dispersive spectroscopy (EDS) confirmed the presence of carbon (C), oxygen (O), nitrogen (N), and zinc (Zn) in the nanocapsules (Figure S1b,c, Supporting Information), further validating our synthesis approach. Subsequent Fourier transform infrared spectroscopy (FT‐IR) analysis (Figure 1e) confirmed the successful attachment of PEG‐Man onto PCNPs, indicated by a peak at 2882 cm⁻¹ corresponding to the C‐H bond stretching vibration. Additionally, the shift of the broad peak from 3403 to 3392 cm⁻¹, attributed to O‐H stretching vibrations in PEG‐Man, suggests the formation of hydrogen bonds between PEG‐Man and PCNPs.^[^ ^22^ ^]^ Moreover, X‐ray photoelectron spectroscopy (XPS) analysis further corroborated the presence of C, N, O, and Zn elements in both PCNPs and PCNPs@PEG‐Man (Figure 1f). A detailed examination of the C1s spectrum revealed a significant increase in the percentage of C‐C bonds from PC and PEG‐Man in PCNPs@PEG‐Man, rising from 15.45% (Figure 1g) to 21.16% (Figure 1h). This finding robustly supports the successful modification of PCNPs with PEG‐Man. Additionally, the elemental ratios of PCNPs and PCNPs@PEG‐Man, as assessed by XPS and EDS, are presented in Figure 1i. Compared to PCNPs, PCNPs@PEG‐Man exhibited increased proportions of C and O elements, while the proportions of N and Zn elements decreased. These results further corroborate the successful modification of nanocapsules with PEG‐Man.

### 2.2 Radical Scavenging Capacity and Gastrointestinal Stability of PCNPs@PEG‐Man

Reactive oxygen species (ROS) can cause oxidative damage to proteins, lipids, and DNA, leading to intestinal inflammation, mucosal damage, and ulcer formation.^[^ ^23^ ^]^ PCNPs nanocapsules, enriched with procyanidins (PC), have demonstrated notable antioxidant properties and effectively scavenged ROS.^[^ ^24^ ^]^ To evaluate the antioxidant activity of PCNPs@PEG‐Man, we conducted four distinct in vitro assays: DPPH, ABTS, FRAP, and FOX. Given the importance of gastrointestinal stability in oral drug delivery, we compared the antioxidant activities of natural vitamin C (VC), PCNPs, and PCNPs@PEG‐Man after 2 h of exposure to simulated gastric fluid (SGF) or simulated intestinal fluid (SIF).

**Figure 2:**
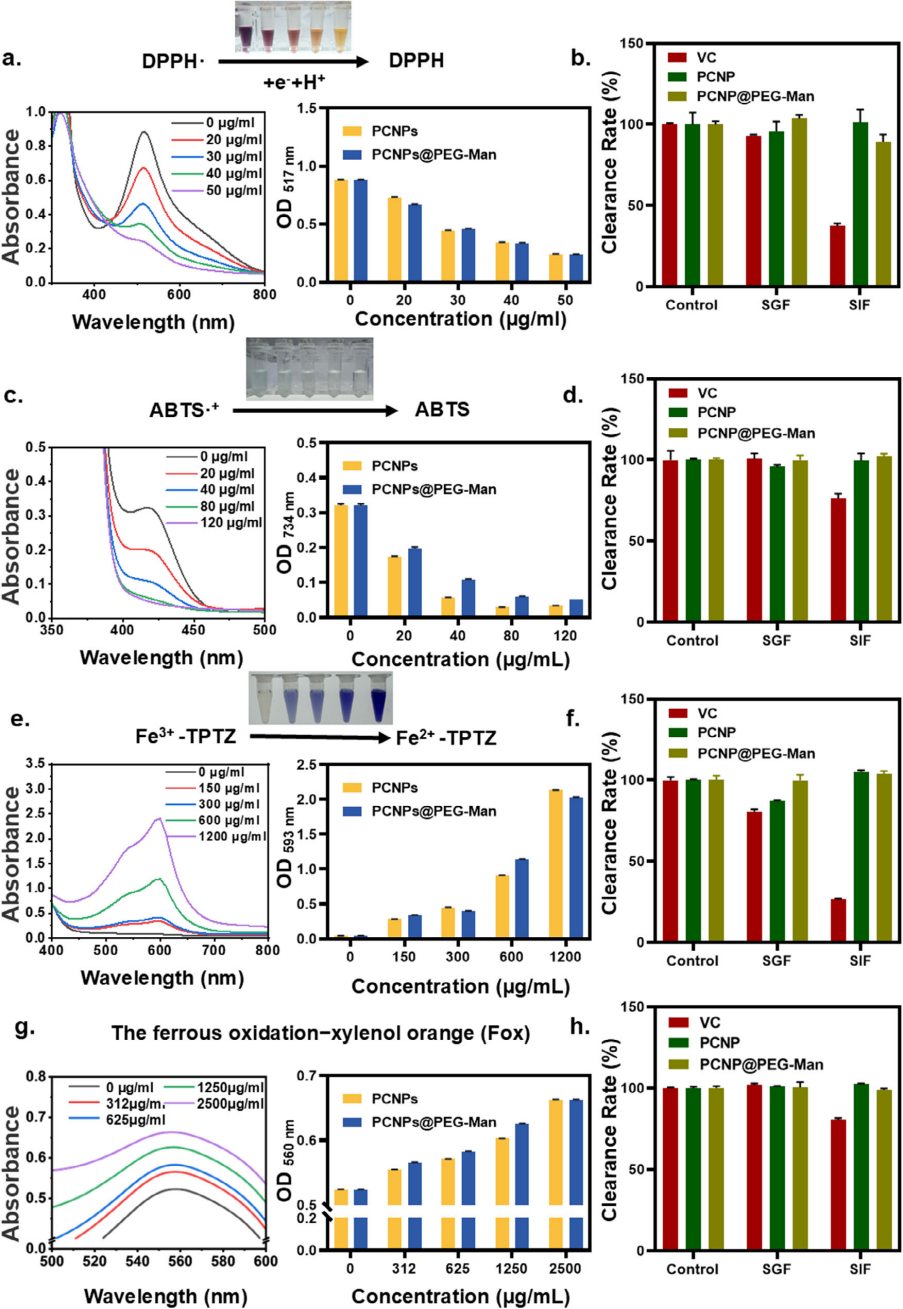
Radical scavenging capacity and gastrointestinal stability of PCNPs@PEG‐Man assessed using various assays, including a,b) DPPH (absorbance at 517 nm), c,d) ABTS (absorbance at 734 nm), e,f) FRAP (absorbance at 593 nm), and g,h) FOX (absorbance at 560 nm). Data are mean ± SD (n = 3).

The DPPH radical, a stable nitrogen‐centered chromogenic species with a distinct absorption peak at 517 nm, exhibited significant radical scavenging activity.^[^ ^25^ ^]^ At a concentration of 50 µg ml^−1^, both PCNPs and PCNPs@PEG‐Man scavenged ≈75% of the radicals, demonstrating comparable antioxidant effects without significant attenuation after modification (**Figure** **2**a; Figure S2a,b, Supporting Information). In terms of gastrointestinal stability, both PCNPs and PCNPs@PEG‐Man were incubated in SGF (pH = 2) or SIF (pH = 7) at 37°C for 2 h. Their antioxidant performance showed minimal change, whereas VC's antioxidant capacity decreased by 7% and 63% after treatment with SGF and SIF, respectively (Figure 2b; Figure S2c, Supporting Information). This indicates that the nanocapsules not only possess excellent antioxidant properties but also exhibit significant tolerance to gastrointestinal conditions, enhancing their potential efficacy in targeting inflammation.

In the ABTS assay, antioxidants inhibit the formation of blue‐green ABTS cation radicals (ABTS^+^), which have a characteristic absorption peak at 734 nm.^[^ ^26^ ^]^ The results showed that 50 µg ml^−1^ of PCNPs scavenged 90% and PCNPs@PEG‐Man scavenged 84% of the radicals, respectively (Figure 2c; Figure S2d,e, Supporting Information). In contrast, the VC group's radical scavenging ability decreased by 14% after 2 h of treatment with SIF, while the antioxidant performance of the nanocapsules showed negligible attenuation (Figure 2d; Figure S2f, Supporting Information).

The FRAP method assessed the reducing capability of samples, where antioxidants convert Fe^3^⁺‐TPTZ to blue Fe^2^⁺‐TPTZ under acidic conditions, with a characteristic absorption peak at 593 nm.^[^ ^27^ ^]^ The results showed that both PCNPs and PCNPs@PEG‐Man exhibited concentration‐dependent antioxidant capacity (Figure 2e; Figure S2g,h, Supporting Information). Compared to the control group, the antioxidant capacity of VC decreased by 20% after 2 h of treatment with SIF, while that of PCNPs decreased by only 12.5%. After 2 h of treatment with SIF, VC's antioxidant capacity by 74% (Figure 2f; Figure S2i, Supporting Information).

The ferrous oxidation−xylenol orange (FOX) assay was used to quantify the remaining unreacted H_2_O_2_ after incubation. Absorption at 560 nm was recorded at 25 °C after combining 200 µL FOX solution with 5 µL of reaction mixtures containing 500 mm H_2_O_2_ and varying concentrations of PCNPs or PCNPs@PEG‐Man over 3 h. The results revealed that the antioxidant capacity of both nanocapsule formulations remained largely unaffected (Figure 2g; Figure S2j.k, Supporting Information). In contrast, the antioxidant capacity of VC decreased by 20% after treatment with SGF and SIF (Figure 2h; Figure S2l, Supporting Information).

To further evaluate the antioxidant efficacy of PCNPs@PEG‐Man in vivo, we conducted additional experiments in a murine colitis model. We assessed systemic and tissue oxidative stress levels by measuring malondialdehyde (MDA) in peripheral blood (Figure S3a, Supporting Information) and performing dihydroethidium (DHE)‐based reactive oxygen species (ROS) immunofluorescence detection in colonic tissues (Figure S4a, Supporting Information).

The MDA analysis revealed that the normal group exhibited an average MDA level of 2.4731 mmol L^−1^. In contrast, DSS‐induced colitis significantly elevated MDA levels to 11.0214 mmol L^−1^. Treatment with PCNPs@PEG‐Man markedly reduced lipid peroxidation, lowering MDA levels to an average of 5.4166 mmol mL^−1^, which closely approximated the levels observed in the healthy group (Figure S3b, Supporting Information). The quantitative DHE immunofluorescence results demonstrated that the ROS levels in the colitis‐positive control group increased to 2.43‐fold compared to the normal group. Both treatment groups exhibited significant recovery, with the PCNPs group showing ROS levels at 1.83‐fold of the normal group, and the PCNPs@PEG‐Man group at 1.15‐fold, which was closest to the oxidative stress levels observed in the normal control group (Figure S4b, Supporting Information).

These findings underscore the antioxidant stability and superiority of PCNPs@PEG‐Man nanocapsules in gastrointestinal environments. Their remarkable resilience to harsh gastrointestinal conditions, combined with effective ROS scavenging capabilities, highlights their potential utility in targeting inflammation within the gastrointestinal tract. Thus, PCNPs@PEG‐Man nanocapsules position themselves as promising candidates for further development in oral drug delivery systems aimed at inflammatory disorders.

### 2.3 Anti‐Oxidative and Anti‐Inflammatory Effects of PCNPs@PEG‐Man at Cellular Level

**Figure 3:**
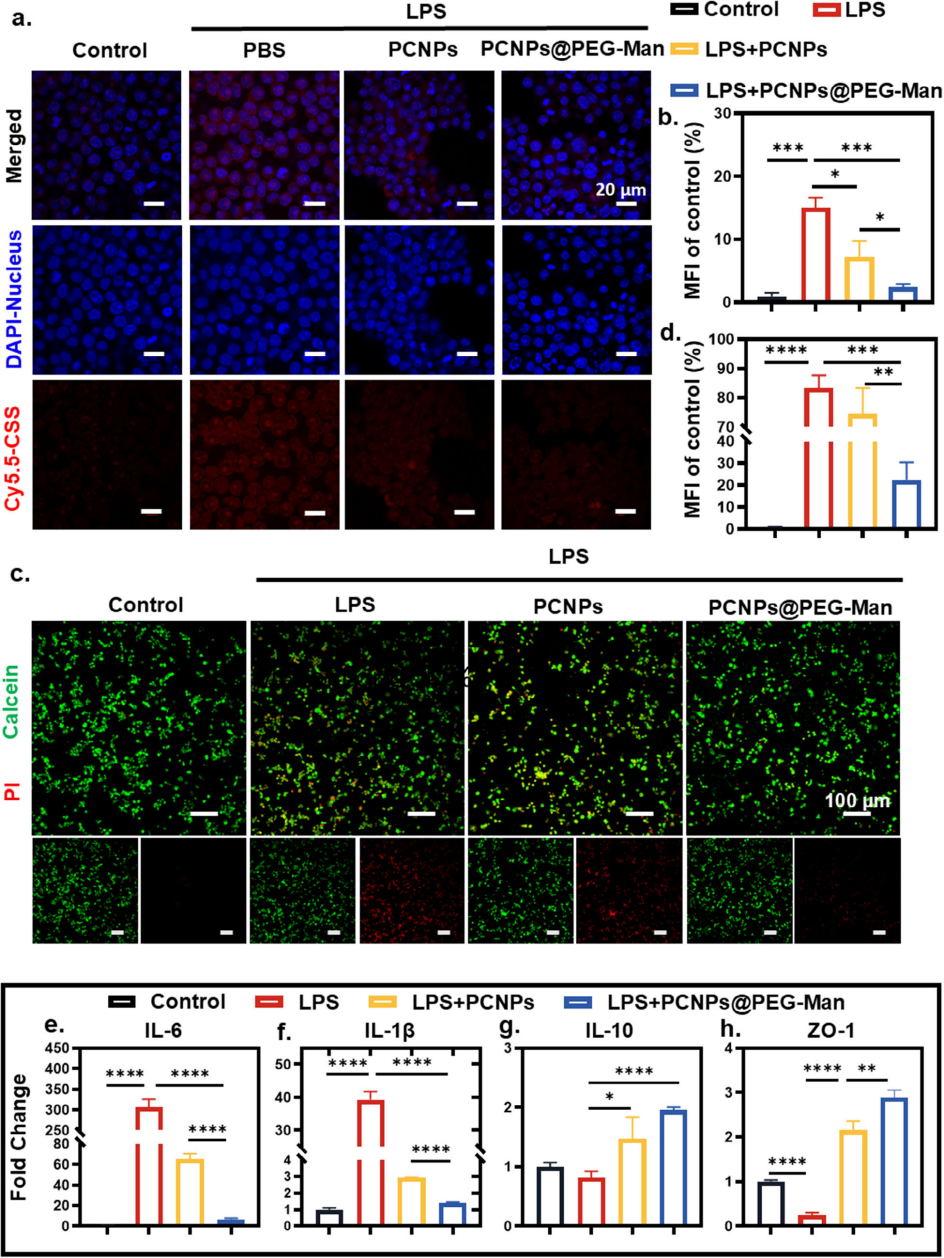
Anti‐oxidative and anti‐inflammatory effects of PCNPs@PEG‐Man at cellular level. a,b) PCNPs@PEG‐Man protected RAW 264.7 cells from the oxidative stress caused by LPS. Scale bar: 20 µm c,d) Live/dead cell staining shows fewer damaged cells in the PCNPs@PEG‐Man group. Scale bar: 100 µm e,f) PCNPs@PEG‐Man decreases pro‐inflammatory cytokines (IL‐6, IL‐1β) and g) increases anti‐inflammatory IL‐10 expression. h) PCNPs@PEG‐Man increases ZO‐1 expression. Data are mean ± SD (n = 3). Statistical analysis was performed using Student's t‐test. *P < 0.05, **P < 0.01, ***P < 0.001, ****P < 0.0001.

To evaluate the potential of PCNPs@PEG‐Man in mitigating oxidative stress and inflammation, we conducted experiments in RAW264.7 macrophages, a well‐established model for studying inflammation.^[^ ^28^ ^]^ Upon stimulation with LPS, there was a significant increase in red fluorescence intensity, indicating elevated ROS levels compared to the control group, confirming that LPS induces oxidative stress. However, treatment with PCNPs and PCNPs@PEG‐Man markedly reduced the ROS fluorescence intensity by 7.26‐fold and 2.43‐fold, respectively, relative to the LPS group. This reduction underscores the superior ROS scavenging ability of PCNPs@PEG‐Man, which nearly restored ROS levels to those observed in untreated control cells (**Figure** **3**a,b), demonstrating its potent antioxidant effects.

Further validation came from live/dead cell staining, which showed a significant reduction in cell damage within the PCNPs@PEG‐Man group, closely mirroring the control. The damage induced by LPS was characterized by a marked increase in red fluorescence intensity, indicative of cellular damage. In contrast, treatment with PCNPs and PCNPs@PEG‐Man substantially reduced the extent of cell damage, with PCNPs@PEG‐Man exhibiting over a three‐fold decrease in damage compared to LPS treatment alone (Figure 3c,d). This result highlights the potential of PCNPs@PEG‐Man to protect cells from oxidative stress‐induced cytotoxicity, reinforcing its anti‐inflammatory capabilities.

In addition to ROS scavenging, PCNPs@PEG‐Man significantly modulated the expression of key pro‐ and anti‐inflammatory cytokines. LPS stimulation notably upregulated the expression of pro‐inflammatory markers IL‐6 and IL‐1β, both of which are critical in mediating the inflammatory response. However, treatment with PCNPs and PCNPs@PEG‐Man resulted in a significant downregulation of these cytokines, with PCNPs@PEG‐Man exhibiting a stronger effect, indicating its enhanced anti‐inflammatory potential. Concurrently, the anti‐inflammatory cytokine IL‐10 was significantly increased following treatment with PCNPs@PEG‐Man, further supporting its role in mitigating inflammation (Figure 3e–g).

Moreover, the expression of ZO‐1, a crucial tight junction protein responsible for maintaining intestinal barrier integrity, was markedly elevated in cells treated with PCNPs and PCNPs@PEG‐Man, compared to the LPS group. The upregulation of ZO‐1 by PCNPs@PEG‐Man suggests its potential to protect the gut barrier, a critical target in inflammatory diseases^[^ ^29^ ^]^ (Figure 3h). These findings also confirm that PCNPs@PEG‐Man demonstrated low cytotoxicity, robust ROS scavenging, and significant anti‐inflammatory activities in vitro, making it a promising candidate for therapeutic strategies aimed at inflammation‐related conditions.

The reduction of oxidative stress and inflammatory cytokine expression, along with the preservation of tight junction proteins, highlights the dual benefits of PCNPs@PEG‐Man in combating both inflammation and oxidative damage. These results are consistent with previous studies that have demonstrated the therapeutic potential of antioxidants and anti‐inflammatory agents in managing gastrointestinal disorders. Future research should focus on exploring the in vivo effects of PCNPs@PEG‐Man, particularly validating its efficacy in animal models of colitis. Such studies should also investigate its impact on gut microbiota and mucosal healing, further elucidating the compound's therapeutic potential.

### 2.4 The Targeting Ability of PCNPs@PEG‐Man in Inflamed Intestine

To evaluate the targeting efficiency and therapeutic potential of PCNPs@PEG‐Man in inflamed tissues, we conducted a series of in vitro and in vivo experiments aimed at investigating the enhanced uptake and retention of these nanocapsules under inflammatory conditions. PCNPs@PEG‐Man demonstrated remarkable endocytic efficiency in Caco‐2 cells, particularly under inflammatory conditions. CLSM imaging revealed that inflammation significantly enhanced the uptake of PCNPs@PEG‐Man capsules by Caco‐2 cells (Figure S5a, Supporting Information). Quantitative analysis indicated that, in the absence of DSS induction, the integrated density per unit area (Int Den/area) for PCNPs was 153.59 ± 23.39, while PCNPs@PEG‐Man showed a significantly higher value of 216.21 ± 11.51 (P < 0.05). In the presence of 5% DSS, which induces an inflammatory state, the Int Den/area value for PCNPs increased to 389.15 ± 46.82, whereas that for PCNPs@PEG‐Man surged to 1120.23 ± 34.53 (P < 0.001), indicating superior uptake by inflamed cells (Figure S5b, Supporting Information).

**Figure 4:**
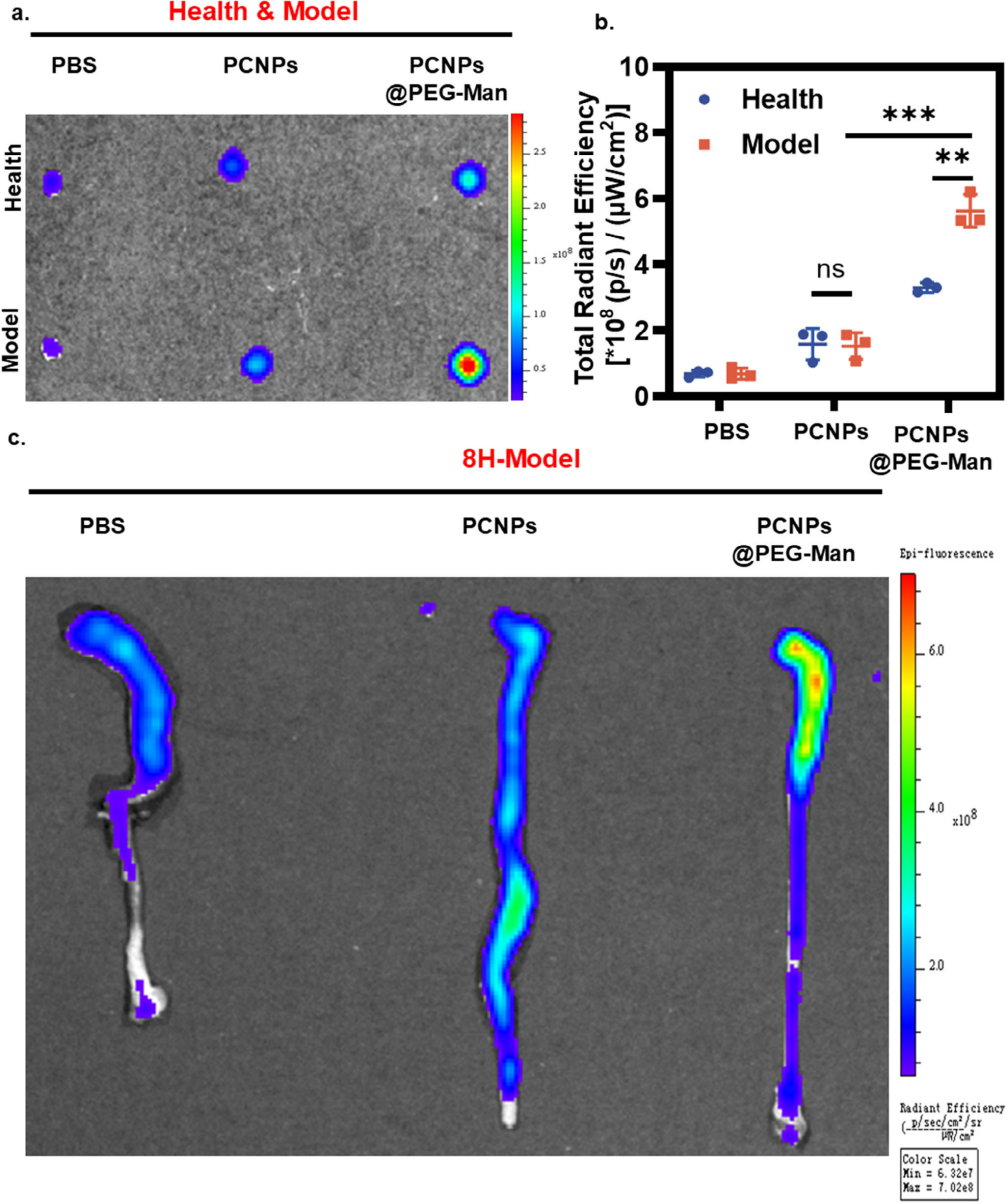
The targeting ability of PCNPs@PEG‐Man in inflamed intestine. a) IVIS images of ex vivo colons from healthy mice or DSS‐induced colitis models after 30 min of incubation with PBS, PCNPs, and PCNPs@PEG‐Man. b) Quantification of the fluorescence intensity in the individual colons in different groups. Ex vivo imaging of tissues from mice orally administered with PBS, PCNPs, or PCNPs@PEG‐Man, c) IVIS images of major visceral organs; d) IVIS images of colonic tissues. Data are mean ± SD (n = 3). Statistical analysis was performed using Student's t‐test. *P < 0.05, **P < 0.01, ***P < 0.001, ****P < 0.0001.

The enhanced adhesion of PCNPs@PEG‐Man to the inflamed colonic epithelium may be attributed to their negative charge, which is hypothesized to interact favorably with the positively charged components of the inflamed tissue. To validate this hypothesis, the nanocapsules were labeled with Rhodamine B (RhB) and imaged ex vivo.^[^ ^30^ ^]^ Results indicated that colons treated with PCNPs@PEG‐Man in the acute colitis model exhibited the highest fluorescence intensity (**Figure** **4**a). Quantitative analysis revealed that the fluorescence intensity of the colon in the healthy group was 0.69 ± 0.06×10^8^, while the fluorescence intensity of the PCNPs group and PCNPs@PEG‐Man group was 1.59 ± 0.28×10^8^ and 3.30 ± 0.09×10^8^. In the acute colitis model group, the fluorescence intensity remained at 0.69 ± 0.14×10^8^, while the PCNPs group and PCNPs@PEG‐Man group exhibited intensities of 1.53 ± 0.33×10^8^ and 5.63 ± 0.40×10^8^, respectively (Figure 4b). The significant increase in fluorescence intensity in the inflamed colon for the PCNPs@PEG‐Man group strongly suggests that mannose modification enhances targeting in inflammation conditions.

Overall, the enhanced targeting ability of PCNPs@PEG‐Man may facilitate localized therapeutic effects in the treatment of IBD by increasing drug concentrations at inflammatory sites while minimizing systemic exposure. The results underscore the potential of utilizing PEGylation and mannose receptor targeting as effective strategies to optimize drug delivery systems for managing colitis and other inflammatory disorders.

To comprehensively evaluate the in vivo performance of mannose‐modified nanocapsules, we conducted a series of experiments in a murine colitis model. Six mice with acute colitis were randomly allocated into three treatment groups receiving PBS, PCNPs, or PCNPs@PEG‐Man via oral gavage. Fluorescence imaging of colonic tissues revealed significantly enhanced accumulation of PCNPs@PEG‐Man at inflammatory sites compared to unmodified PCNPs (Figure 4c), demonstrating the targeting advantage conferred by mannose modification.

To further characterize the temporal biodistribution profile, we performed longitudinal fluorescence imaging of nanocapsule localization in the gastrointestinal tract. While both formulations showed comparable retention during the initial 6 h period, PCNPs@PEG‐Man exhibited superior persistence at 12 h post‐administration, with a 1.51‐fold higher fluorescence intensity than unmodified PCNPs (Figure S6, Supporting Information). This extended retention suggests that mannose‐mediated targeting not only improves initial accumulation but also prolongs nanocapsule residence time in inflamed tissues.

These findings collectively demonstrate that the dual strategy of PCNPs@PEG‐Man significantly enhances both the spatial specificity and temporal persistence of nanocapsule delivery to colonic inflammation sites. The improved targeting efficiency of PCNPs@PEG‐Man holds particularly promise for IBD treatment by extending the retention time of drugs in the colon. This approach may represent an effective platform for developing targeted therapies against colitis and other inflammatory conditions where localized drug delivery is critical.

### 2.5 Targeting Ability of PCNPs@PEG‐Man to Mannose Receptors

IBD is characterized by remitting and relapsing inflammation within the intestinal mucosa and lamina propria. In this context, macrophages are recognized as critical targets for interventional strategies aimed at modulating inflammatory responses.^[^ ^31^ ^]^ To determine whether the enhanced uptake of PCNPs@PEG‐Man was due to its specific targeting of mannose receptors on macrophages, a series of competitive binding and cellular uptake studies were conducted.

**Figure 5:**
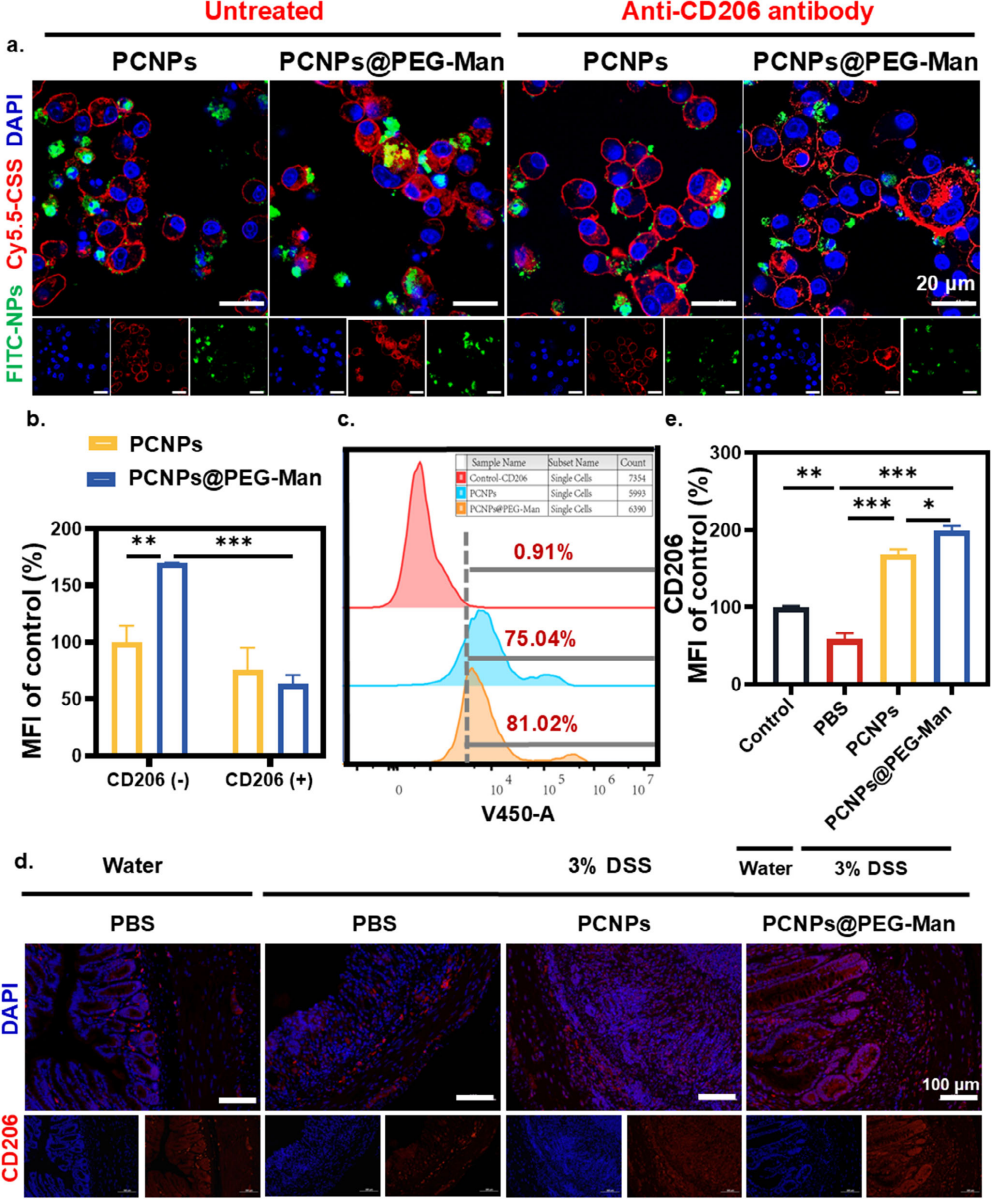
PCNPs@PEG‐Man's mannose receptor targeting. a,b) anti‐CD206 antibody pre‐treatment significantly reduces PCNPs@PEG‐Man uptake, confirming mannose receptor‐mediated internalization. Scale bar: 20 µm c) Flow cytometry shows increased CD206 expression in RAW 264.7 cells after PCNPs@PEG‐Man treatment. d,e) Immunofluorescence staining and quantitative analysis of CD206 expression in inflamed colon tissues. Scale bar: 100 µm. Data are mean ± SD (n = 3). Statistical analysis was performed using Student's t‐test. *P < 0.05, **P < 0.01, ***P < 0.001, ****P < 0.0001.

RAW 264.7 cells were pre‐treated with anti‐CD206 antibody, a competitive inhibitor of mannose receptors, for 3 h, followed by a 4‐h incubation with either PCNPs or PCNPs@PEG‐Man. Control cells were incubated with PCNPs without prior anti‐CD206 antibody treatment. After 4 h, fluorescence intensity measurements revealed that the uptake of PCNPs@PEG‐Man was ≈1.70 ± 0.005 times higher than that of the control group. However, in the presence of anti‐CD206 antibody, the uptake of PCNPs@PEG‐Man significantly diminished, resulting in no notable difference in fluorescence intensity between the PCNPs and PCNPs@PEG‐Man groups. Notably, fluorescence was predominantly localized around the cell membrane under competitive inhibition conditions, suggesting limited internalization of nanocapsules (**Figure** **5**a,b). These findings robustly indicate that the cellular uptake of PCNPs@PEG‐Man is mediated via mannose receptors.

Further analysis using flow cytometry revealed that treatment with PCNPs@PEG‐Man markedly upregulated the expression of CD206 in RAW 264.7 cells, increasing from 0.91% in the control condition to 81.02% (Figure 5c). This observation underscores that incubation with PCNPs@PEG‐Man significantly upregulates the expression of mannose receptors on macrophages, thereby enhancing the ability of 4arm‐PEG‐Man‐modified nanocapsules to bind to and internalize via CD206 receptors on the surface of RAW 264.7 cells.

**Figure 6:**
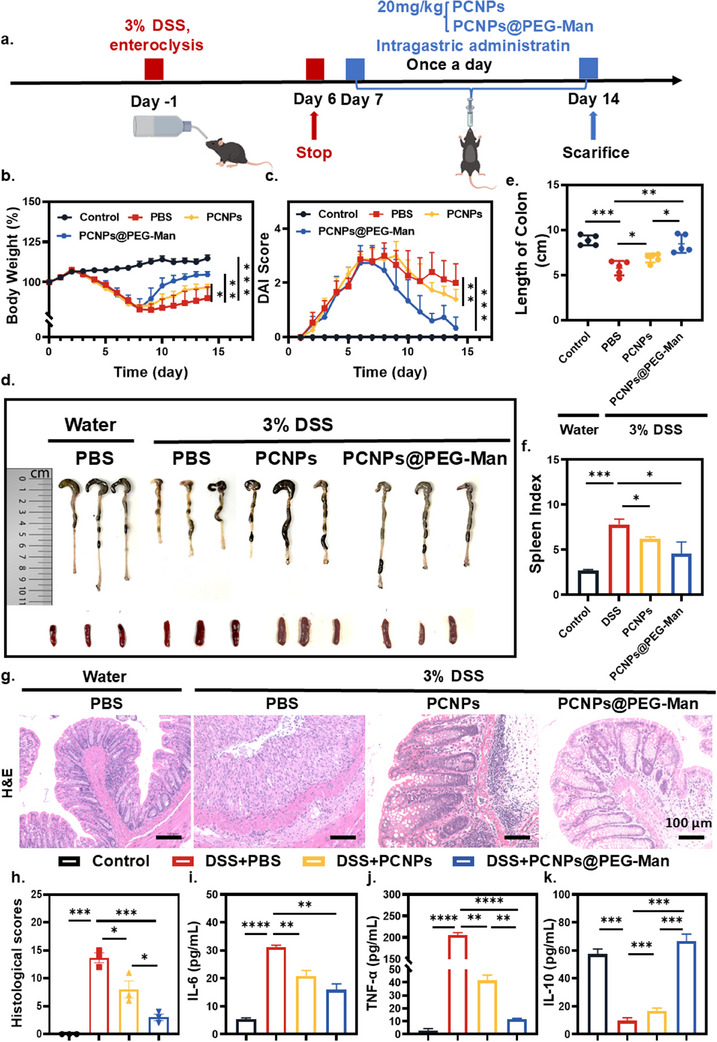
Therapeutic efficacy of PCNPs@PEG‐Man in a mice model with DSS‐induced acute colitis. a) The experimental design for colitis treatment. Mice were fed with 3% DSS for 1 week to develop colitis and then the mice were daily administrated with 20 mg kg^−1^ of PCNPs and PCNPs@PEG‐Man by oral gavage for another 7 days. PBS was used as a control. Then the mice were euthanized under deep anesthesia and colons were harvested for subsequent analysis. b) Variation of body weight during infection and treatment for 14 d. c) Daily disease activity index (DAI) score changes in each group. d) Digital photos of cecum‐colon tissues and representative macroscopic spleen appearance. e) The length of colon tissues harvested after treatment (n = 5). f) The changes in spleen index in different groups. g) Representative histological images of colon tissuesstained with hematoxylin and eosin (H&E) from 3 biologically independent animals in each group. Scale bar: 100 µm h) Histopathology scores of colon tissues based on H&E images. i–k) The serum concentration of (i) IL‐6, (j) TNF‐𝛼, and (k) IL‐10 detected by the ELISA kit (n = 3). Data are mean ± SD. Statistical analysis was performed using Student's t‐test. *P < 0.05, **P < 0.01, ***P < 0.001, ****P < 0.0001.

Additionally, the dual‐targeting capability of PCNPs@PEG‐Man should not be limited to in vitro studies using RAW264.7 cells. To address this, we have not only examined the expression of CD206 receptors in RAW264.7 macrophages but also conducted immunofluorescence staining of colonic tissues from colitis model mice to systematically analyze the expression levels of mannose receptors (CD206) in the colonic tissues of these mice. Importantly, Immunofluorescence staining revealed a significant upregulation of CD206 fluorescence intensity in the PCNPs@PEG‐Man treatment group, exhibiting a 1.99 ± 0.08‐fold increase compared to the control group (Figure 5d,e). This enhanced CD206 expression indicates a macrophage phenotypic transition from the pro‐inflammatory M1 state to the anti‐inflammatory M2 state, facilitating inflammation resolution and tissue repair. This is consistent with the IL‐10 levels observed in qPCR (Figure 3g) and ELISA (Figure 6k) results regarding inflammatory factors and indicates that the PCNPs@PEG‐Man group exhibited reduced inflammation levels, with macrophages predominantly shifting toward an anti‐inflammatory phenotype. Collectively, these results demonstrate that PCNPs@PEG‐Man effectively suppresses inflammatory responses, promotes anti‐inflammatory cytokine production, and enhances macrophage polarization toward a reparative M2 phenotype, highlighting its therapeutic potential for inflammation‐associated conditions.In conclusion, our study demonstrates that PCNPs@PEG‐Man specifically targets mannose receptors on inflammatory macrophages, significantly enhancing their uptake in inflamed environments. This targeted delivery not only improves the efficacy of nanocapsules but also offers a novel therapeutic strategy for mitigating inflammation. The dual mechanism of action—targeting and reprogramming macrophages—offers a promising approach for enhancing treatment outcomes in IBD.

### 2.6 Therapeutic Effect of PCNPs@PEG‐Man on DSS‐Induced Colitis Model Mice

After confirming the enhanced mucosal adhesion ability of PCNPs@PEG‐Man under pathological conditions and obtaining promising in vivo results, we further investigated their potential as therapeutic agents for improving acute colitis. For this purpose, we utilized DSS‐induced colitis mice, a well‐established animal model for IBD, as illustrated in the experimental scheme in **Figure** **6**a. The mice in the DSS‐treated group exhibited a series of symptoms, such as weight loss and higher DAI scores, together with a shortened colon and damaged epithelial cells, suggesting the successful onset of colitis.^[^ ^32^ ^]^

Notably, mice treated with PCNPs@PEG‐Man demonstrated significant weight gain compared to those receiving PBS or PCNPs alone, which failed to effectively prevent weight loss (Figure 6b). The DAI further corroborated these findings, revealing a significant reduction in disease activity in the PCNPs@PEG‐Man‐treated group (Figure 6c; and Table S1, Supporting Information). These results suggest that PCNPs@PEG‐Man not only enhances mucosal adhesion but also contributes to improved clinical outcomes in colitis.

On Day 14, following euthanasia under anesthesia, colon tissues were collected to assess length and analyze major visceral organs (heart, liver, spleen, lung, kidney).

The colon images show that PCNPs@PEG‐Man treatment significantly preserved colon length, unlike the noticeable shortening seen in the PBS and PCNPs groups (Figure 6d,e). Additionally, The spleen index indicated that the PCNPs@PEG‐Man group experienced a notably accelerated resolution of inflammation, as evidenced by a reduced spleen index (Figure 6f). These findings strongly indicate that PCNPs@PEG‐Man effectively mitigates systemic inflammation associated with colitis.

Histological analysis via H&E staining revealed pronounced inflammatory features in DSS‐treated mice, including mucosal erosion, goblet cell destruction, and inflammatory cell infiltration. To quantitatively assess the extent of colonic tissue damage, histological scores were calculated according to established guidelines (scoring criteria provided in Table S4, Supporting Information). The PCNPs group exhibited moderate mucosal infiltration and goblet cell damage. In contrast, the PCNPs@PEG‐Man nanocapsules markedly reversed colonic inflammation, restored mucosal architecture, and reduced inflammatory cell infiltration (Figure 6g). The histological scores supported these observations: the DSS group exhibited scores ranging from 12‐15, the PCNPs group ranged from 6‐11, while the PCNPs@PEG‐Man group exhibited the most favorable therapeutic outcomes, with scores ranging from 2‐4, closely aligning with the normal group^[^ ^33^ ^]^ (Figure 6h).

To elucidate the underlying mechanisms of the observed therapeutic effects, we employed ELISA kits to measure the concentrations of pro‐inflammatory cytokines (IL‐6, TNF‐α) and the anti‐inflammatory cytokine IL‐10 in serum samples. As anticipated, treatment with PCNPs@PEG‐Man resulted in a marked decrease in the secretion of pro‐inflammatory cytokines (IL‐6, TNF‐α) (Figure 6i,j) and a substantial increase in IL‐10 secretion (Figure 6k). These findings underscore the anti‐inflammatory efficacy of PCNPs@PEG‐Man in colitic mice, suggesting a mechanism through which these nanocapsules modulate immune responses and enhance tissue healing.

In summary, our results suggest that PCNPs@PEG‐Man nanocapsules effectively prevent and mitigate the severity of colitis, presenting a promising therapeutic approach for managing IBD.

### 2.7 Restorative Effect of PCNPs@PEG‐Man on Intestinal Barrier

Tight junctions (TJs) are important component of the intestinal epithelial barrier, regulating its selective permeability to prevent pathogenic antigens in the intestinal lumen from entering the mucosal lamina propria, which can trigger intestinal and systemic inflammation and immune responses.^[^ ^34^ ^]^ These TJs are complex structures composed of various proteins, including transmembrane proteins, peripheral membrane proteins, and cytoskeletal proteins.^[^ ^35^ ^]^ Among them, Occludin is a key transmembrane protein that regulates TJ permeability and maintains cell polarity.^[^ ^36^ ^]^ Zonula occludens (ZOs), as peripheral membrane proteins, contribute to the integrity of the TJ complex by linking claudins, occludin, and cytoskeletal proteins.^[^ ^37^ ^]^

**Figure 7:**
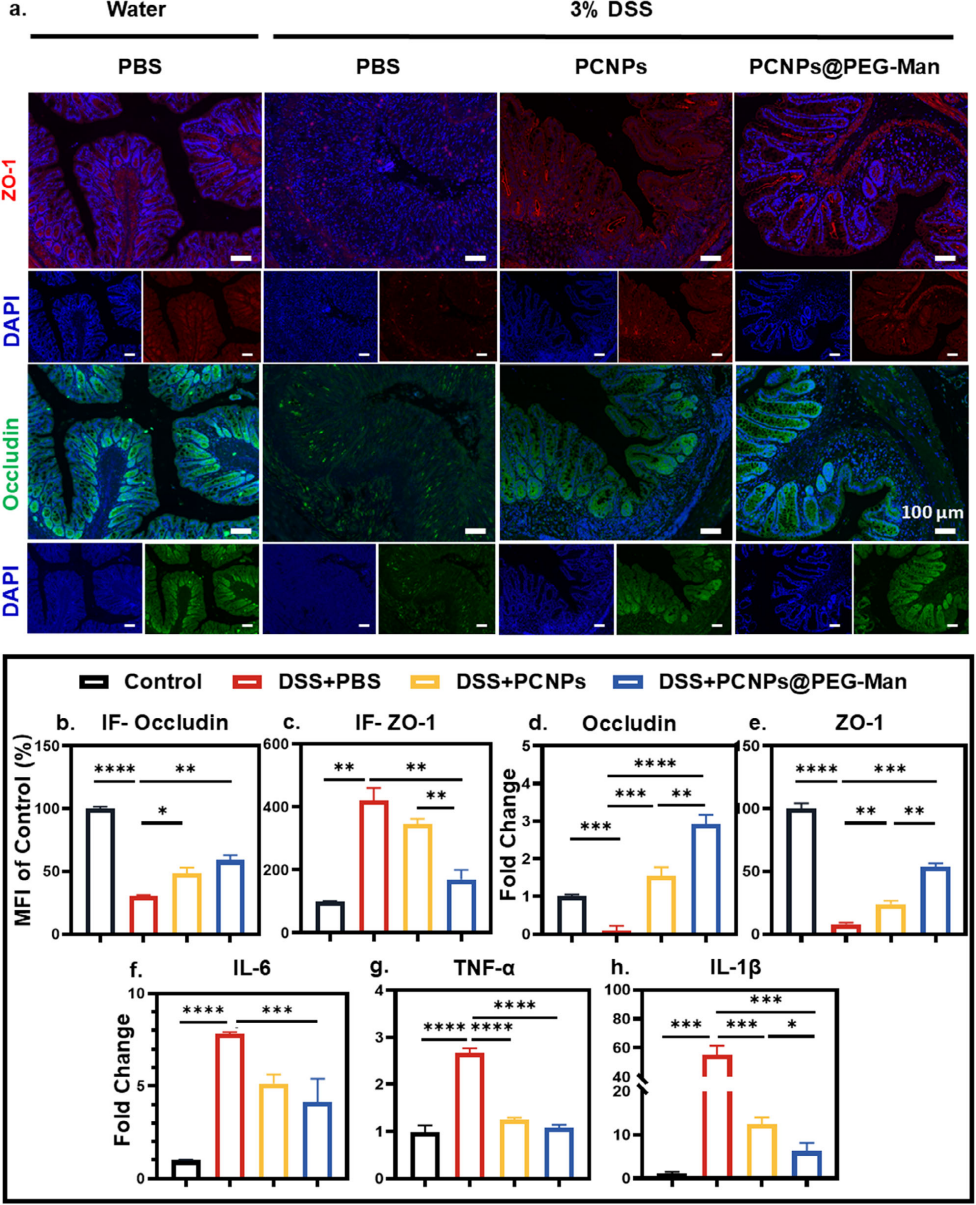
Treatment efficacy of PCNPs@PEG‐Man against DSS‐induced intestinal barrier damage. a) Tight‐junction proteins staining to evaluate intestinal barrier damage. Scale bar: 100 µm. Semiquantitative analysis of the expression of b) occludin and c) ZO‐1 in the colon based on immunofluorescence staining. d–h) Expression of tight junction proteins (d) Occludin and (e) ZO‐1 and pro‐inflammatory cytokines ((f) IL‐6, (g) TNF‐α, and (h) IL‐1β) in colon tissues detected by RT‐PCR. Data are mean ± SD (n = 3). Statistical analysis was performed using Student's t‐test. *P < 0.05, **P < 0.01, ***P < 0.001, ****P < 0.0001.

Furthermore, immunofluorescence analysis revealed a deficiency in intestinal tight junction proteins in IBD mice. however, PCNPs@PEG‐Man significantly mitigated the damage to intestinal barrier integrity induced by DSS, such as tight junction proteins (ZO‐1 and Occludin) (**Figure** **7**a–c). These findings suggest that PCNPs@PEG‐Man effectively restores the function of tight junctions, thereby enhancing the protective capabilities of the intestinal barrier.

To further elucidate the therapeutic potential of PCNPs@PEG‐Man, we analyzed homogenates from colon tissues using reverse transcription and RT‐PCR. The results indicated that, compared to DSS‐treated mice, levels of tight junction proteins (ZO‐1 and occludin) were significantly increased in PCNPs@PEG‐Man‐treated mice, while pro‐inflammatory cytokines (TNF‐α, IL‐6, and IL‐1β) were notably reduced (Figure 7d–h). This reduction in pro‐inflammatory cytokines highlights the anti‐inflammatory effects of PCNPs@PEG‐Man and underscores its potential as a therapeutic agent for acute colitis.

Overall, these findings collectively indicate that PCNPs@PEG‐Man not only enhances the expression of tight junction proteins but also reduces inflammation, demonstrating remarkable therapeutic efficacy in DSS‐induced mouse models of acute colitis.

### 2.8 Biosafety Profiles of PCNPs@PEG‐Man

PCNPs@PEG‐Man has demonstrated significant anti‐inflammatory and pro‐resolution biological effects, indicating its potential as an effective therapeutic agent for colitis. However, before advancing to potential clinical applications, a comprehensive evaluation of the biocompatibility of PCNPs@PEG‐Man is essential.

**Figure 8:**
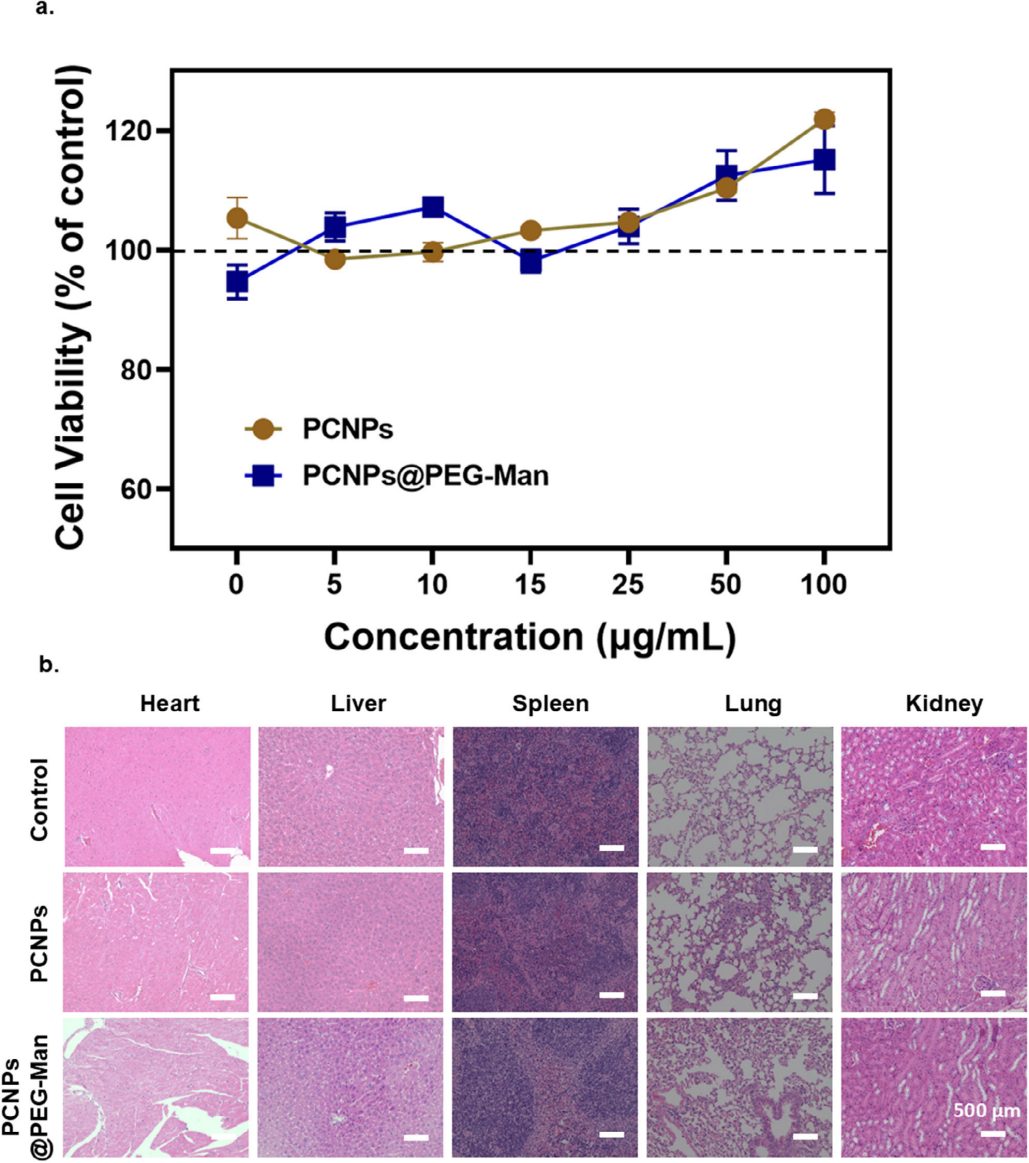
Biocompatibility evaluation of PCNPs@PEG‐Man. a) Cell viability of RAW 246.7 cells incubated with various concentrations of PCNPs@PEG‐Man for 24 h (data represent mean ± SD, n = 6) b) H&E staining, scale bar: 500 µm.

To assess the cytotoxic effects of PCNPs@PEG‐Man, we performed CCK‐8 assays using RAW 264.7 macrophage cells. The results indicated that cell viability remained unaffected at concentrations up to 100 µg ml^−1^ (**Figure** **8**a). This finding suggests that PCNPs@PEG‐Man exhibits low cytotoxicity, making it a promising candidate for therapeutic use in inflammatory conditions.

Histological analysis of major organs, including the heart, liver, spleen, lung, and kidney, was conducted to evaluate any potential systemic effects of PCNPs@PEG‐Man. According to the scoring criteria (Table S2, Supporting Information), the main internal organs are all scored 0, indicating no organic changes compared to those of non‐administered mice (Figure 8b). These findings indicate favorable biosafety profiles and negligible side effects associated with the administration of PCNPs@PEG‐Man. In conclusion, the comprehensive evaluation of biocompatibility in this study suggests that PCNPs@PEG‐Man possesses a favorable safety profile.

### 2.9 Regulation of the Gut Microbiota by PCNPs@PEG‐Man in Colitic Mice

Accumulating evidence indicates that the gut microbiome plays a pivotal role in the progression of IBD.^[^ ^38^ ^]^ Given the promising therapeutic effects of PCNPs@PEG‐Man in colitis, we investigated whether this treatment could reshape the dysbiosis in the gut microbiota of a colitis mouse model, potentially halting disease progression. To assess the impact of PCNPs@PEG‐Man on the gut microbiota, we employed 16S rDNA amplicon sequencing, utilizing various diversity metrics for amplicon sequence variants analyzed through QIIME 2.^[^ ^39^ ^]^

**Figure 9:**
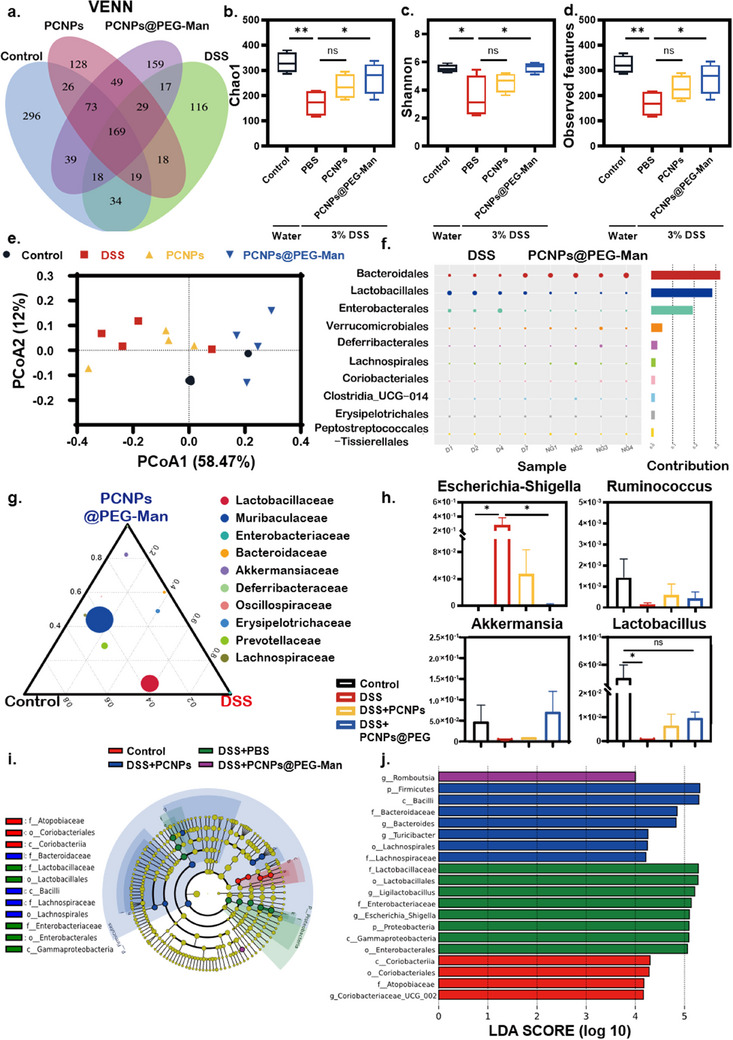
Modulation of gut bacteria by PCNPs@PEG‐Man during colitis treatment. a) Venn diagram of bacterial species in each group. Comparison of alpha diversity assessed by b) Chao1 index, c) Shannon index, and d) observed species. e) Principal coordinates analysis (PCoA) showed the β‐diversity of the gut microbiome. f) Simper analysis showing the relative abundance of order. g) Effects of PCNPs@PEG‐Man on microbial composition at family levels using ternary plot method. h) Changes in the presence of four intestinal bacteria genera in different groups i) Cladogram based on Linear discriminant analysis effect size (LEfSe) analysis showing community composition of the gut microbiota in mice. j) Distribution histogram based on linear discriminant analysis (LDA). LDA score higher than 3 indicates a higher relative abundance in the corresponding group than that in other groups. LDA (log10) > 4.0, P < 0.05. Data are mean ± SD (n = 4). Statistical analysis was performed using Student's t‐test. *P < 0.05, **P < 0.01, ***P < 0.001, ****P < 0.0001.

The Venn diagram (**Figure** **9**a) illustrates the shared or distinct operational taxonomic units (OTUs) among groups. The normal group exhibited 674 OTUs, while the DSS group had only 420 OTUs. The PCNPs group presented 511 OTUs, and the PCNPs@PEG‐Man group had 553 OTUs, with all four groups sharing 169 OTUs. The 𝛼 diversity indices, namely, the Chao1 index, Shannon index, and observed species, were estimated to ascertain the richness and diversity of the bacterial composition. It is well‐documented that that DSS‐induced colitis is always accompanied by a decline in microbiome diversity and community abundance.^[^ ^40^ ^]^ Higher bacterial diversity correlates with better host health.^[^ ^41^ ^]^ Notably, the PCNPs@PEG‐Man group exhibited OTU richness closest to that of the normal mice, signifying a substantial improvement in gut microbiota diversity. Specifically, the Chao1 index, Shannon index, and observed species were significantly elevated in the PCNPs@PEG‐Man treatment group compared to the other groups, underscoring the efficacy of PCNPs@PEG‐Man in preventing the reduction of microbial diversity in colitis mice (Figure 9b–d).

Furthermore, principal coordinate analysis (PCoA) was used to evaluate differences in microbial diversity between samples^[^ ^42^ ^]^ (Figure 9e). PCoA, based on weighted UniFrac distances, revealed that mice in the PCNPs@PEG‐Man treatment group formed a distinct cluster compared to the DSS‐induced colitis group. This distinction was not observed in the PCNPs group, which might be attributed to the superior targeting ability of the mannose modification. To quantify the contribution of each species to the observed differences between groups, we performed SIMPER analysis, which decomposes the Bray‐Curtis dissimilarity index. At the order level, Figure 9f illustrates that the contribution of Bacteroidales was diminished in the DSS group, reflecting microbial imbalance, while its contribution increased in the PCNPs@PEG‐Man treatment group, suggesting a rebalancing of the gut microbiome.

To identify specific bacterial taxa and compare microbial composition across groups, we employed a ternary plot (Figure 9g) to highlight the most abundant taxa at the family level. Muribaculaceae, a family within the Bacteroidales order, is crucial for producing short‐chain fatty acids (SCFAs) from both endogenous (mucin glycans) and exogenous polysaccharides (dietary fibers).^[^ ^43^ ^]^ This family engages in cross‐feeding interactions with probiotic genera such as Bifidobacterium and Lactobacillus.^[^ ^44^ ^]^ Both the normal and PCNPs@PEG‐Man groups maintained similar levels of Muribaculaceae. Additionally, we observed that Escherichia‐Shigella, known for its pro‐inflammatory properties and disruption of gut barrier integrity,^[^ ^45^ ^]^ was significantly elevated in the DSS group but diminished in both the PCNPs and PCNPs@PEG‐Man groups, returning to levels comparable to the normal group. Furthermore, the abundances of Ruminococcus (whose reduction is associated with pathogenic bacterial overgrowth and gut inflammation),^[^ ^46^ ^]^ Akkermansia (which enhances the thickness and integrity of the gut mucus layer),^[^ ^47^ ^]^ and Lactobacillus (known for its anti‐inflammatory properties and role in regulating gut microbiota and strengthening the gut barrier)^[^ ^48^ ^]^ were markedly reduced in the DSS group but restored in the treatment groups (Figure 9h). These results indicate that PCNPs@PEG‐Man has a beneficial effect on restoring key gut microbiota in colitis mice.

Notably, Roseburia, a symbiotic bacterium that produces SCFAs exhibits anti‐inflammatory properties and regulates colonic motility.^[^ ^49^ ^]^ The LDA distribution bar chart demonstrated that Roseburia was significantly enriched in the PCNPs@PEG‐Man group compared to the other groups (Figure 9i,j), suggesting that the superior anti‐inflammatory effects of PCNPs@PEG‐Man may be partially attributed to the enrichment of this beneficial bacterium. This observation is particularly relevant, as butyrate is known to provide energy to colonocytes, enhance gut barrier function, and exert systemic anti‐inflammatory effects.

These findings collectively highlight the potential of PCNPs@PEG‐Man to alleviate colitis symptoms while simultaneously restoring a balanced and diverse gut microbiome, which is crucial for maintaining intestinal health and preventing disease progression. The capacity to reshape the gut microbiota may thus represent a pivotal mechanism underlying the therapeutic efficacy of PCNPs@PEG‐Man.

## 3 Discussion and Conclusion

Oxidative stress and gut microbiota dysbiosis form a self‐perpetuating cycle throughout the progression of inflammatory bowel disease (IBD).^[^ ^50^ ^]^ Excessive reactive oxygen species (ROS) exacerbate intestinal barrier dysfunction by directly impairing epithelial integrity and triggering inflammatory cascades.^[^ ^51^ ^]^ Simultaneously, the oxidative microenvironment selectively suppresses the colonization of symbiotic anaerobes while promoting the proliferation of pro‐inflammatory microbial species, thereby aggravating microbial dysbiosis.^[^ ^52^ ^]^ This reciprocal interplay indicates that alleviating oxidative stress could create a supportive niche for beneficial microbiota, while targeted microbiota modulation may, in turn, reduce oxidative damage.

Therefore, we have successfully developed an intestinal dual‐targeted oral carrier‐free nanocapsule, PCNPs@PEG‐Man, for the therapy of IBD. This formulation effectively modulates the gut microbiota and disrupts the detrimental cycle of ROS and inflammation. The PC in PCNPs@PEG‐Man plays a crucial role in ROS scavenging, exhibiting anti‐inflammatory and antioxidant properties that promote the restoration of redox balance. The negatively charged mannose‐modified PCNPs@PEG‐Man could not only accurately target the site of colonic inflammation through electrostatic interactions, but could also be effectively endocytosed by macrophages through mannose receptor‐mediated endocytosis. Results from 16S rDNA gene sequencing demonstrated that PCNPs@PEG‐Man significantly increased microbial diversity in the intestines by reducing the abundance of pathogens such as Escherichia‐Shigella and enhancing the levels of beneficial probiotics, including Ruminococcus, Acromyces, and Lactobacillus. Moreover, PCNPs@PEG‐Man facilitated the repair of the intestinal epithelial barrier by upregulating tight junction proteins ZO‐1 and Occludin. It also regulated levels of pro‐inflammatory cytokines (TNF‐α, IL‐6, IL‐1β) to within normal ranges and promoted the reprogramming of macrophages to the M2 phenotype, demonstrating good biosafety in an enteritis model. This therapeutic effect stems from the dual biological roles of PC: acting as a substrate for microbial metabolism to generate bioactive metabolites that support probiotic proliferation,^[^ ^53^ ^]^ and exhibiting selective antimicrobial activity against dysbiosis‐associated pathogens through biofilm disruption and virulence suppression.^[^ ^54^ ^]^

Overall, PCNPs@PEG‐Man represents a novel carrier‐free nanocapsule for IBD therapeutics, combining dual‐targeted delivery with anti‐inflammatory, antioxidant and intestinal microbiota regulation effects. This dual‐pronged treatment strategy provides new ideas for developing targeted drug delivery systems for IBD, particularly suited to complex pathological conditions requiring simultaneous management of oxidative damage and microbial dysbiosis.

## 4 Experimental Section

### Materials

DMEM (Gibco, USA), Zinc nitrate hexahydrate (ZnNO₃, Macklin), procyanidin (PC, Macklin), 1,2‐dimethylimidazole (C₄H₆N₂, Aladdin), 4‐PEG‐Man (Ruixibio); Hydrochloric acid (HCl, Aladdin), Hydrogen peroxide solution (H₂O₂, Aladdin), Xylenol orange indicator (Aladdin), Pepsin powder (Yuanye Bio‐Technology), Total antioxidant capacity assay kit (Beyotime Biotechnology), Calcein /PI cell viability/CytotoxicityAssay kit (Beyotime Biotechnology), Cell Counting Kit‐8 (CCK‐8, Dojindo Molecular Technologies), Trypsin from porcine pancreas (Sigma Aldrich), DPPH radical scavenging assay kit (Solarbio Science & Technology), ABTS radical scavenging assay kit (Solarbio Science & Technology), Lipopolysaccharide (LPS, Sigma‐Aldrich), dextran sulfate sodium (DSS, MeilunBio), ELISA kits for IL‐10, IL‐6, and TNF‐α (Solarbio Science & Technology), Brilliant Violet 421™ anti‐mouse CD206 antibody (BioLegend, UK), Red fluorescent reactive oxygen species (ROS) detection kit (Applygen), Anti‐fade mounting medium containing DAPI (Biosharp), FastPure Cell/Tissue Total RNA Isolation Kit V2 (Vazyme), HiScript II Q RT SuperMix for qPCR (Vazyme), Hieff qPCR SYBR Green Master Mix (No Rox, Yeasen), Actin‐Tracker Red‐Rhodamine (Beyotime Biotechnology), cell culture dishes/plates (NEST Biotechnology Co. Ltd.), Malondialdehyde (MDA) Content Assay Kit (Solarbio Science & Technology).

### Preparation of PCNPs

Weigh 0.432 g of 1,2‐dimethylimidazole (MIM) and divide into two portions, each dissolved in 1.52 ml deionized water. While stirring at 800 rpm, add 76 µL of 0.6 M Zn(NO₃)₂·6H₂O to each portion. Centrifuge at 12,000 rpm for 15 min, discard the supernatant, and resuspend the pellet in 500 µL deionized water. Gradually add the suspension to 4.25 ml of 20 mg ml^−1^ PC solution and stir at 600 rpm for 30 min. Centrifuge at 15,000 rpm for 15 min, discard the supernatant, and wash the capsules 3–4 times with deionized water. Divide the capsules into three portions, treat with 200 µL of 5 mm EDTA for 1 h, and denucleate at 15,000 rpm for 15 min. Repeat 2 times, lyophilize 100 µL for quantification, and store the remainder at 4°C.

### Preparation of PCNPs@PEG‐Man

Dissolve 5 mg of 4‐PEG‐Man in deionized water and add the above PCNPs, making up the volume to 10 ml. Stir the mixture at 600 rpm for 30 min The following day, centrifuge at 15,000 rpm for 15 min and wash the capsules 3 times. Divide the modified and unmodified capsules into three portions, each with 200 µL of 5 mm EDTA, and denucleate for 1 h. Centrifuge at 15,000 rpm for 15 min, repeat 1 to 2 times, lyophilize 100 µL into powder for quantification, and store the rest at 4°C for further use.

### Instruments

Transmission electron microscopy (TEM, FEI Talos F200S) was used to observe the morphology of nanocapsules. Confocal laser scanning microscopy (CLSM) installed on a Nikon A1 system was used to study cell micrographs. Optical microscope images were taken using a NIKON Ni‐U microscope with a 10x bright field lens. Fourier‐transform infrared spectroscopy (FTIR, Bruker TENSOR II) was used to measure the infrared spectra of nanocapsules and modified materials. X‐ray photoelectron spectroscopy (XPS) was obtained on a Thermo Electron ESCALAB 250 spectrometer. Ultraviolet‐visible (UV–vis) spectra were measured using a CARY 5000 UV‐Vis‐NIR spectrophotometer (USA). Sample absorbance was detected using a Lambda 25 spectrophotometer (PerkinElmer, USA). CytoFLEX flow cytometer (Beckman Coulter, USA), Vortex mixer (Bioland, China), Roche LightCycler 480 fluorescence quantitative PCR instrument (Roche), and IVIS Lumina XRMS Series III (PerkinElmer, USA) were also used.

### Radical Scavenging Assay

In the present experiments, PCNPs and PCNPs@PEG‐Man were comparatively evaluated using various antioxidant assays, including the ferric reducing antioxidant power (FRAP), PTIO^•‐^scavenging, ABTS^•‐^scavenging, DPPH^•‐^scavenging assays and FOX‐scavenging assays. Simulated gastric (SGF) and intestinal fluid (SIF) was used to mimic the in vivo pH environments.^[^ ^55^ ^]^ The changes in antioxidant capacity of VC solution, PCNPs, and PCNPs@PEG‐Man capsules after soaking in SGF and SIF for 2 h were compared, and the absorbance was recorded according to the instruction of the kits.

### Cell Culture

Mouse monocytic macrophage leukemia cells (RAW 264.7) and human colon adenocarcinoma cells (Caco‐2) were obtained from the American Type Culture Collection (ATCC). The cells were cultured in Dulbecco's Modified Eagle Medium (DMEM) supplemented with 10% heat‐inactivated fetal bovine serum (FBS), 1% penicillin (5000 U ml^−1^), and 1% streptomycin (5000 µg ml^−1^). All cell cultures were maintained at 37 °C in a humidified incubator with 5% CO₂ atmosphere.

### CCK‐8 Cell Viability Assay

Cell viability was assessed using the standard Cell Counting Kit‐8 (CCK‐8) method. Caco‐2 or RAW 264.7 cells were seeded in 96‐well plates (1 × 10⁴ per well). After cell adhesion, different concentrations of nanocapsules (0, 5, 10, 15, 25, 50, and 100 µg mL^−1^) were added and incubated at 37 °C for 24 h. The cells were then washed three times with PBS buffer and incubated with 10% CCK‐8 solution (100 µL) at 37°C for 2 h. Finally, cell viability was determined by measuring absorbance at 450 nm.

### LPS‐Induced Inflammatory Stimulation

To induce an inflammatory response, the cells were treated with 1 µg mL^−1^ lipopolysaccharide (LPS) for 6 h under standard culture conditions at 37 °C in a 5% CO₂ atmosphere.^[^ ^56^ ^]^ Following LPS stimulation, cells were washed with PBS and subjected to further treatments.

### CLSM Imaging and Quantitative Analysis

After treatment, cells were fixed with 4% paraformaldehyde for 15 min at room temperature and washed three times with PBS. The fixed cells were then permeabilized using 0.1% Triton X‐100 for 10 min, followed by another set of PBS washes. Nuclei were counterstained with Anti‐fade mounting medium containing DAPI.

Imaging was performed using a CLSM with oil immersion objectives. Fluorescence intensity from at least 3 randomly selected fields per sample was captured and analyzed using Image J software (version 1.5.3, National Institutes of Health, USA). Quantitative analysis of fluorescence intensity was conducted to evaluate cellular uptake and localization.

### In Vitro ROS Scavenging Assay

After LPS‐induced inflammatory stimulation in RAW 264.7 macrophages (as described in section“LPS‐Induced Inflammatory Simulation”), PCNPs and PCNPs@PEG‐Man capsules were added. Following 24 h of incubation, the supernatant was discarded, and the cells were stained with a red fluorescent ROS detection kit for 30 min. The cells were then processed for CLSM as described in the section “CLSM Imaging and Quantitative Analysis”.

### Calcein/PI Cell Viability/Cytotoxicity Assay

After LPS‐induced inflammatory stimulation in RAW 264.7 macrophages (described in section “LPS‐Induced Inflammatory Stimulation”), PCNPs and PCNPs@PEG‐Man capsules were added. The Calcein AM/PI working solution (1:100) was applied after 24 h of incubation. And the mixture was then cultured at 37°C for another 30 min. Subsequently, the cells were washed three times with PBS and fixed with 4% paraformaldehyde for 15 min. The cells were then processed for CLSM as outlined in the section “CLSM Imaging and Quantitative Analysis”.

### Evaluation of The Targeted Adhesion to The Inflamed Colon

Colitis in mice was chemically induced by administering 3% (w/v) DSS in drinking water for 7 days. Initially, to assess the inflammation‐targeted capacity of PCNPs@PEG‐Man, these capsules were co‐incubated with freshly collected colons from healthy or colitic mice. Specifically, the distal colons of both healthy and colitis‐afflicted mice were harvested and incubated for 30 min with with PBS, PCNPs or PCNPs@PEG‐Man (100 µg mL^−1^), respectively. After incubation, the samples were rinsed with PBS and imaged using IVIS.

For in vivo distribution studies, mice with colitis were orally administered 200 µL of either PCNPs or PCNPs@PEG‐Man (20 mg kg^−1^). Mice were then imaged at 8 h post‐administration using IVIS. Following the imaging, the mice were euthanized, and major organs (heart, liver, spleen, lungs, and kidneys) along with the large and small intestinal colon were collected for further imaging.

### In Vitro Cellular Uptake of PCNPs@PEG‐Man

Caco‐2 cells were seeded in 24‐well polystyrene plates (1 × 10⁵ per well) with round coverslips. Once the cells had adhered, two groups were cultured in fresh medium containing 5% DSS for 24 h, while the other two served as controls. Depending on their group assignment, FITC conjugated PCNPs and FITC conjugated PCNPs@PEG‐Man (50 µg mL^−1^) were added to the respective wells, followed by a 1 h incubation. Cells were stained with Actin‐Tracker Red‐Rhodamine for 30 min after fixed and then processed as described in Section “CLSM Imaging and Quantitative Analysis” for CLSM.

### Antibody Pre‐Incubation and PCNPs@PEG‐Man Endocytosis in Macrophage Model

After LPS‐induced inflammatory stimulation in RAW 264.7 macrophages (as described in section “LPS‐Induced Inflammatory Stimulation”). One group of cells was pre‐incubated with anti‐CD206 blocking antibody for 4 h to inhibit the interaction between CD206 and mannose, while the other groups served as controls. FITC conjugated PCNPs and FITC conjugated PCNPs@PEG‐Man (50 µg mL^−1^) were added according to their group assignment and cultured for an additional hour. Cells were stained with Actin‐Tracker Red‐Rhodamine for 30 min after fixed and subsequently processed as described in Section “CLSM Imaging and Quantitative Analysis” for CLSM.

### Flow Cytometry Analysis

RAW 264.7 macrophages were collected from both normal and LPS‐containing medium, then washed twice with PBS and fixed in 4% paraformaldehyde for 15 min. After fixation, cells were washed again with PBS, and a 100 µL cell suspension was transferred in flow cytometry tubes. Permeabilization was performed using 0.5% Triton X‐100 for 5 min, followed by an additional wash with PBS wash. To block non‐specific binding sites, the cells were incubated with 5% BSA for 30 min, after which they were washed once more with PBS.

Next, Brilliant Violet 421™ anti‐mouse CD206 antibody was added at a 1:20 dilution and incubated in the dark at 4 °C for 30 min. Following the staining, the cells were washed twice with PBS and filtered through a 300 mesh to collect them in flow cytometry tubes. Fluorescence intensity was measured with a CytoFLEX flow cytometer (Beckman Coulter, USA), collecting data from at least 10,000 cells per sample. Data analysis was conducted using FlowJo software, which allowed for the selection of single‐cell populations while excluding debris and dead cells through scatter plots. The proportion of CD206‐positive cells was determined using a 405 nm laser and compared to the untreated control group.

### DSS‐Induced Mouse Acute Colitis Model

To ensure statistical reliability while minimizing unnecessary animal use, 5 mice per group were used for all in vivo experiments, including disease activity index (DAI) monitoring and histopathological analysis. This sample size was determined based on power calculations and previous studies in similar experimental models.^[^ ^57^ ^]^

The DSS solution was freshly prepared daily. Throughout the experiment, the disease activity index (DAI) was monitored daily by measuring weight loss, stool consistency, and fecal occult blood. The acute colitis model was induced by water‐mediated administration of DSS as previously described.^[^ ^58^ ^]^ To induce acute colitis, male C57BL/6J mice were exposed to 3% (w/v) DSS in their drinking water for 7 consecutive days, following a 1‐week acclimatization in a pathogen‐free environment.^[^ ^59^ ^]^ DAI scoring was based on weight loss (0: <1%, 1: 1–5%, 2: 5–10%, 3: 10–18%, 4: >18%), fecal occult blood (0: none, 1–2: mild, 3: moderate, 4: severe), and stool consistency (0: normal, 1: semi‐formed, 2: semi‐unformed, 3: very soft, 4: diarrhea) (Table S1, Supporting Information).

C57BL/6 mice (6–8 weeks old, weighing 22–23 g) were sourced from Beijing Vital River Laboratory Animal Technology Co., Ltd. and housed on a standard diet. The study was conducted in accordance with the guidelines approved by the Animal Ethics Committee of the Wenzhou Institute, University of Chinese Academy of Sciences (Approval No. WIUCAS24072201).

### Biosafety Evaluation

To assess the potential side effects of oral administration of PCNPs@PEG‐Man, mice were administered 200 µL of PCNPs@PEG‐Man (20 mg kg^−1^) daily via oral gavage for a period of 5 days. Following the treatment, the mice were euthanized under deep anesthesia. Tissue samples from the heart, liver, spleen, lungs, and kidneys were collected, with healthy mice serving as controls. The biosafety of the treatment was further evaluated through histological analysis. All tissue samples were preserved in 4% paraformaldehyde (1 mL per sample), and subsequent hematoxylin‐eosin (H&E) staining was performed (scoring criteria in Table S2, Supporting Information).

### Spleen Index Calculation

The spleen index was determined to evaluate the effect of treatments on spleen size, often correlated with inflammation or immune response. The calculation was performed as follows: Mice were euthanized, and their spleens were carefully excised. The wet weight of each spleen was measured using a precision balance to the nearest milligram. The body weight of each mouse was recorded prior to euthanasia. And the spleen index was calculated using the formula:

(1) $$\mathrm{Spleen}\;\mathrm{Index}=\frac{SpleenWeight\left(mg\right)}{BodyWeight\left(g\right)}\times 100\%$$

### Measurements for Inflammatory Cytokines in the Serum

Serum inflammation‐associated factors were detected by ELISA kits including IL‐6, TNF‐α, and IL‐10 according to the manufacturer's instructions.

### Inflammatory Model and qRT‐PCR Analysis of Macrophages and Colonic Tissue

After LPS‐induced inflammatory stimulation in RAW 264.7 macrophages, PCNPs and PCNPs@PEG‐Man were added, and the cells were co‐incubated for 24 h. Total RNA was extracted using the FastPure Cell/Tissue Total RNA Isolation Kit V2, and the concentration and purity of the RNA were assessed using a Nanodrop 2000 spectrophotometer. cDNA synthesis was performed using HiScript II Q RT SuperMix. Quantitative real‐time PCR (qRT‐PCR) was conducted with Hieff^®^ qPCR SYBR Green Master Mix (No Rox) to quantify the mRNA expression of key inflammatory cytokines (IL‐6, IL‐1β, IL‐10, and TNF‐α) and tight junction proteins (ZO‐1and Occludin).

Similarly, distal colon tissue samples (20 mg) from DSS‐induced colitis mice were homogenized using a tissue grinder (Servicebio, KZ‐III‐FP) for RNA extraction. The RNA was reverse transcribed into cDNA, and qPCR was performed to assess the expression levels of inflammatory markers and tight junction proteins. The mRNA levels were normalized to GAPDH, and relative expression was calculated using the 2^−ΔΔCt^ method. Primer sequences are provided in Table S3 (Supporting Information).

### Immunofluorescence

The distal segments of the colon were initially fixed in 4% paraformaldehyde and then sliced into serial sections. To prepare the slides, deparaffinization was performed using xylene, after which the sections were rehydrated through a series of increasing ethanol concentrations. For antigen retrieval, the sections were heated in citrate buffer (0.01 M, pH 6.0, 0.05% Tween‐20) using a steamer at 95°C for 20 min. After antigen retrieval, the sections were treated with 3% hydrogen peroxide in methanol for 10 min to eliminate endogenous peroxidase activity. The slides were then washed three times with Tris‐buffered saline (TBS) before being permeabilized and blocked with 10% normal goat serum in 0.3% Triton X‐100 PBS for 1 h at room temperature.

For immunofluorescence staining, tissue sections were first incubated with primary antibodies, followed by the appropriate conjugated secondary antibodies, and then the colon sections were incubated overnight at 4 °C. After staining, coverslips were mounted using an antifade reagent containing DAPI. Images of the stained sections were captured using CLSM. The primary antibodies employed in this study included anti‐occludin (Abcam, 1:100), anti‐ZO‐1 (Abcam, 1:100), and anti‐CD206 (Abcam, 1:100).

### Gut Microbiota Analysis

16S rRNA gene amplicon sequencing was performed on the Illumina NovaSeq 6000 platform. The Raw data were processed using Qiime 2 (Version 202006), which included reducing sequencing and PCR errors, denoising with DADA2, and generating amplicon sequence variants (ASVs) for taxonomic analysis. Taxonomic classification of ASVs was performed using a naive Bayesian classifier based on the Silva database. Alpha diversity was characterized using the Chao 1 index and Shannon index, while beta diversity was analyzed using principal coordinate analysis (PCoA) based on weighted UniFrac distance matrices.

### Statistical Analysis

All biological experiments were repeated, and the results presented in this paper were representative. Statistical analysis was conducted using GraphPad Prism 8 software, with quantitative data expressed as the mean or mean ± standard deviation. Differences between groups were assessed using Student's t‐test, with P < 0.05 considered statistically significant. Significance markers: ∗P < 0.05, ∗∗P < 0.01, ∗∗∗P < 0.001, ∗∗∗∗ P < 0.0001.

## Conflict of Interest

The authors declare no conflict of interest.

## Author Contributions

Y.C. and L.Z. performed conceptualization. Y.C., K.H., and Z.J. performed data curation. X.Z., L.Z., and J.Y. performed funding acquisition. Y.C., Y.G., and W.W. performed methodology. Y.C. and Y.G. performed project administration. Y.C., K.H., and Z.J. performed software. Y.C. performed wrote the original draft. J.Y., L.Z., and Y.L. performed wrote, reviewed, and edited the draft.

## Supporting information



Supporting Information

Supplemental Data 1

## Data Availability

The data that support the findings of this study are available from the corresponding author upon reasonable request.
