# Supplementary material for: Carrier‐Free Nanocapsule with Dual‐Target Capacity for Synergistically Restoring Inflammatory Microenvironment and Microbiota Dysbiosis in Colitis
Source: Adv Sci (Weinh). 2025 Jul 12;12(38):e00001. doi: 10.1002/advs.202500001 (PMC12520492; doi:10.1002/advs.202500001)
Supplement: Supplementary file 1 — Supporting Information [file ADVS-12-e00001-s001.docx]

**Supporting Information**

**Carrier‐Free Nanocapsule with Dual‐Target Capacity for Synergistically Restoring Inflammatory Microenvironment and Microbiota Dysbiosis in Colitis**

Yingjie Chen ^¶^, Yuhan Gao ^Ʈ, ╫^, Kaiyuan Huo ^Ʈ, ╫^, Zhaofan Jin ^Ʈ^, Wenchao Wang*^ф^*, Xingjie Zan ^Ʈ^, Yanlong Liu ^¶, ♀ *^, Limeng Zhu ^Ʈ *^, and Jianfeng Yang ^Ѱ *^

^¶^ Cixi Biomedical Research Institute, Wenzhou Medical University, Ningbo, Zhejiang 315300, China.

^Ʈ^ Wenzhou Institute, University of Chinese Academy of Sciences, Wenzhou, Zhejiang 325001, China.

^╫^ Postgraduate training base Alliance of Wenzhou Medical University, Wenzhou, Zhejiang 325000, China

*^ф^* School of Pharmacy, Wenzhou Medical University, Wenzhou, Zhejiang 325035, China

^♀^ School of Mental Health, Wenzhou Medical University, Wenzhou, Zhejiang 325000, China.

^Ѱ^ Hangzhou First People's Hospital, Hangzhou, Zhejiang 310000, China

* Corresponding authors. Emails: [yangjianfeng@hospital.westlake.edu.cn](mailto:yangjianfeng@hospital.westlake.edu.cn) (J.Yang); [zhulimeng@ucas.ac.cn](mailto:zhulimeng@ucas.ac.cn) (L. Zhu); [benjaminlyl@wmu.edu.cn (Y](mailto:benjaminlyl@wmu.edu.cn%20(Y). Liu);


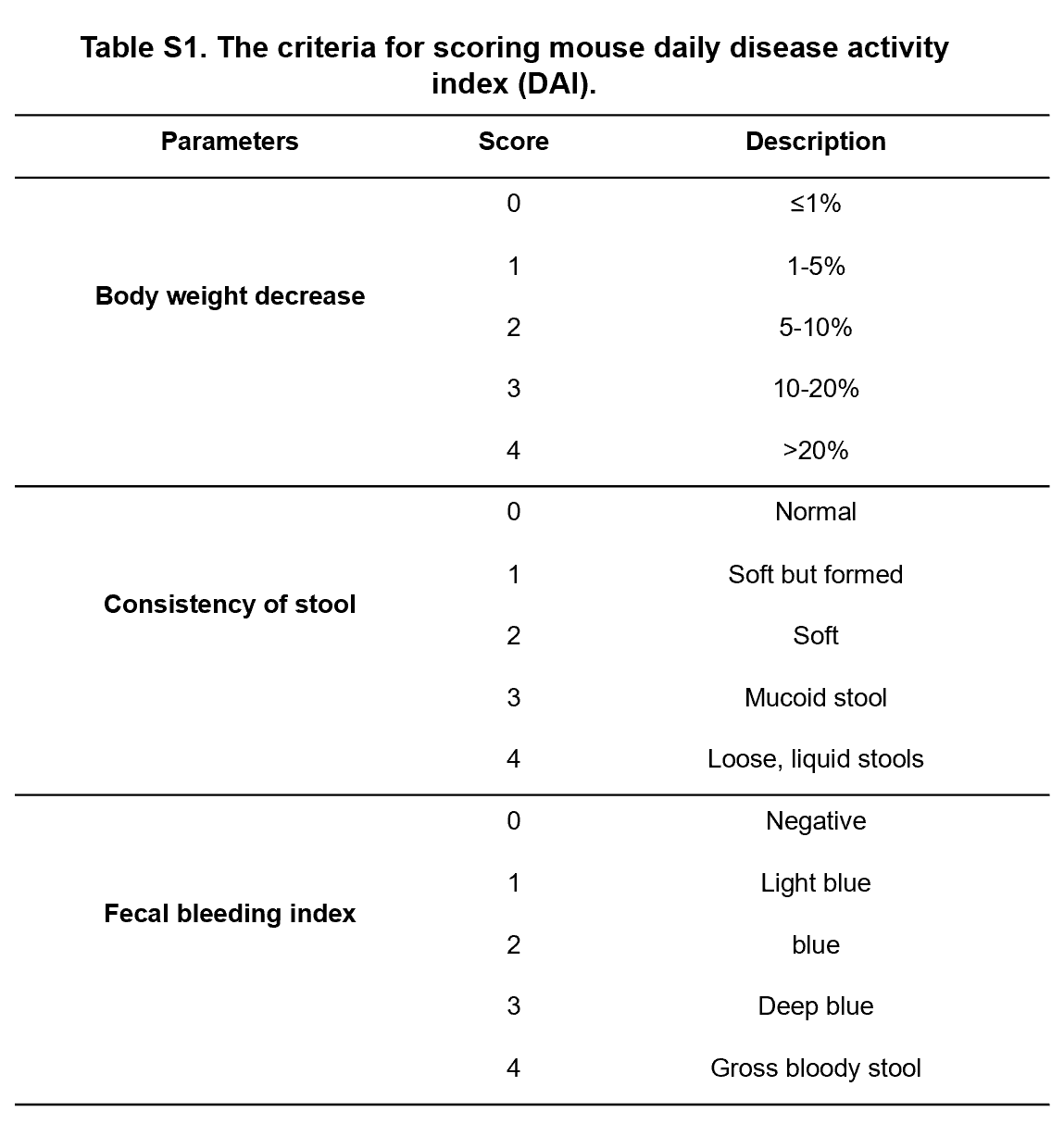


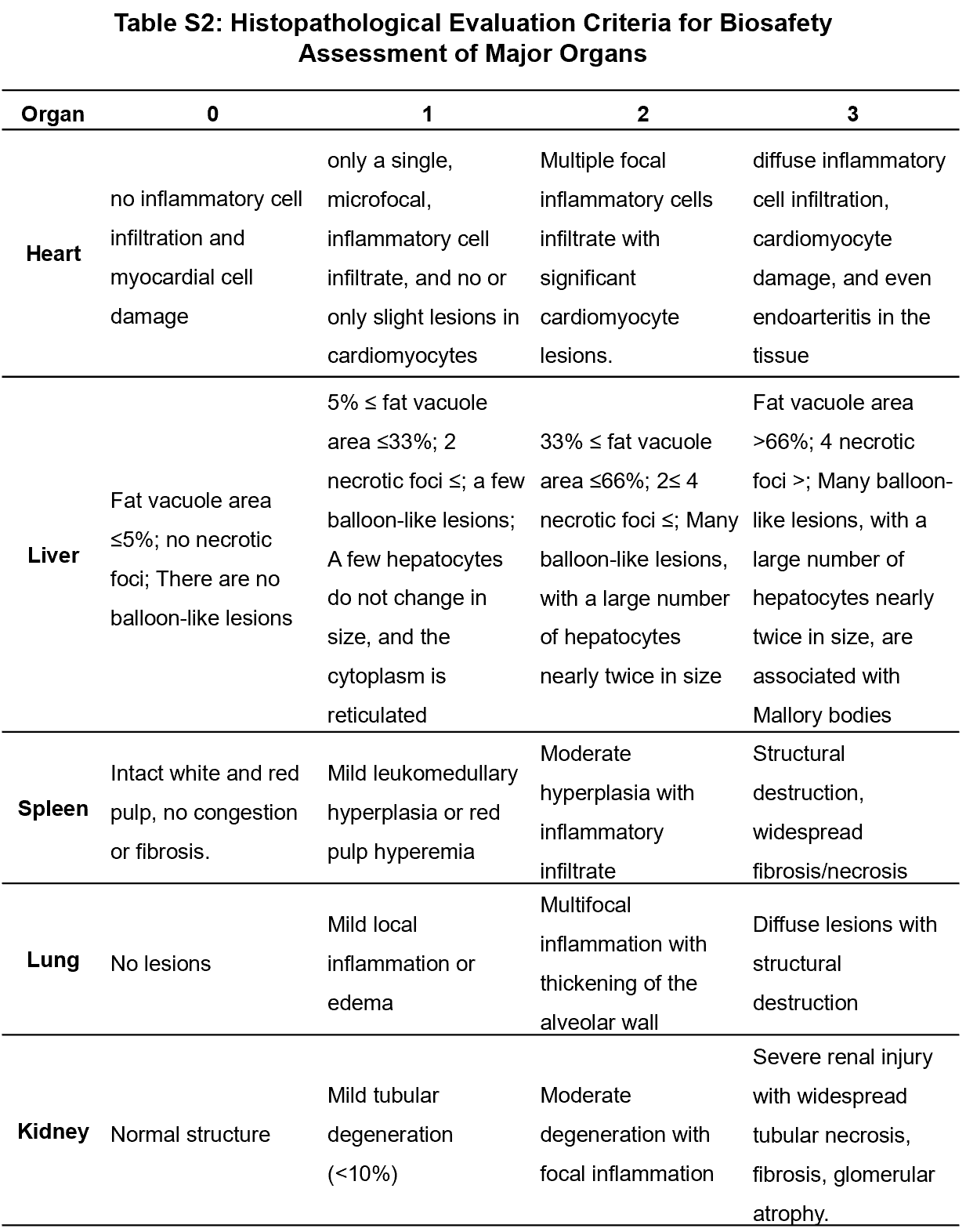


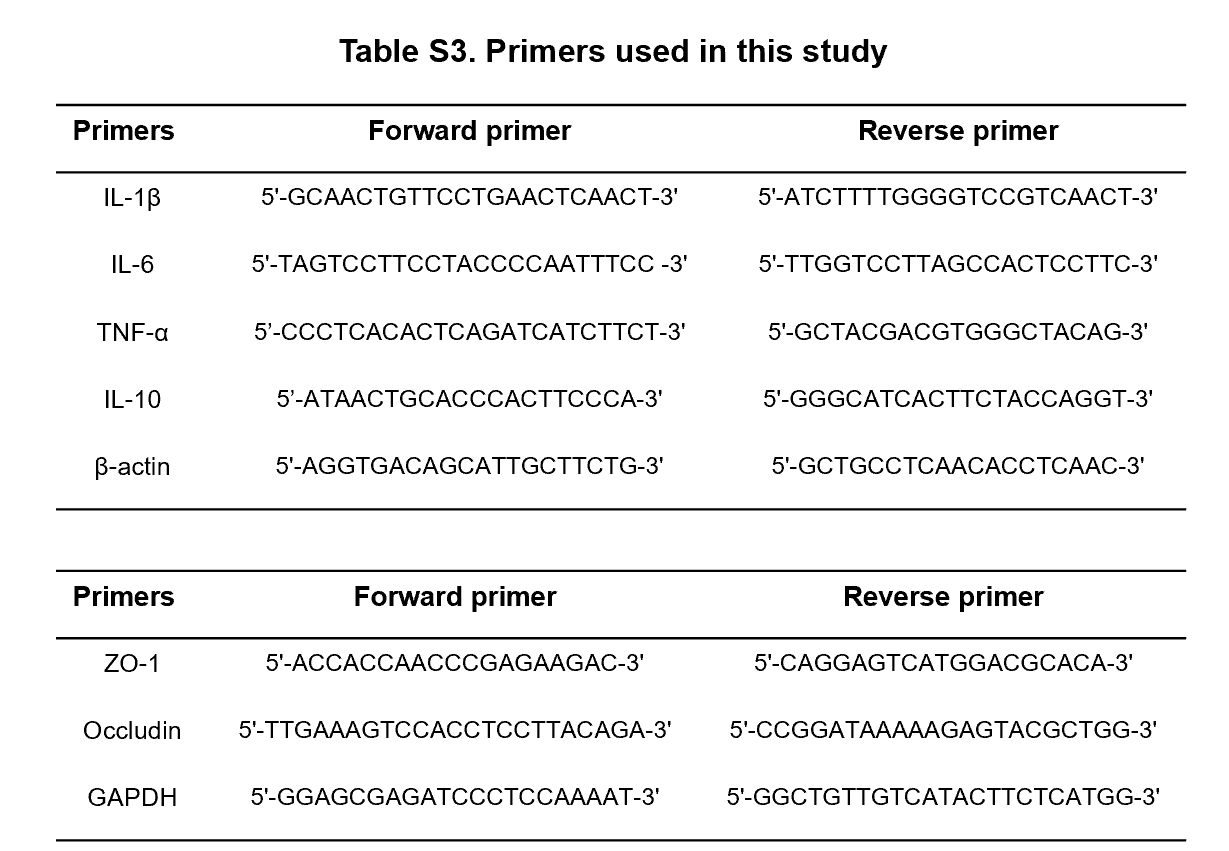

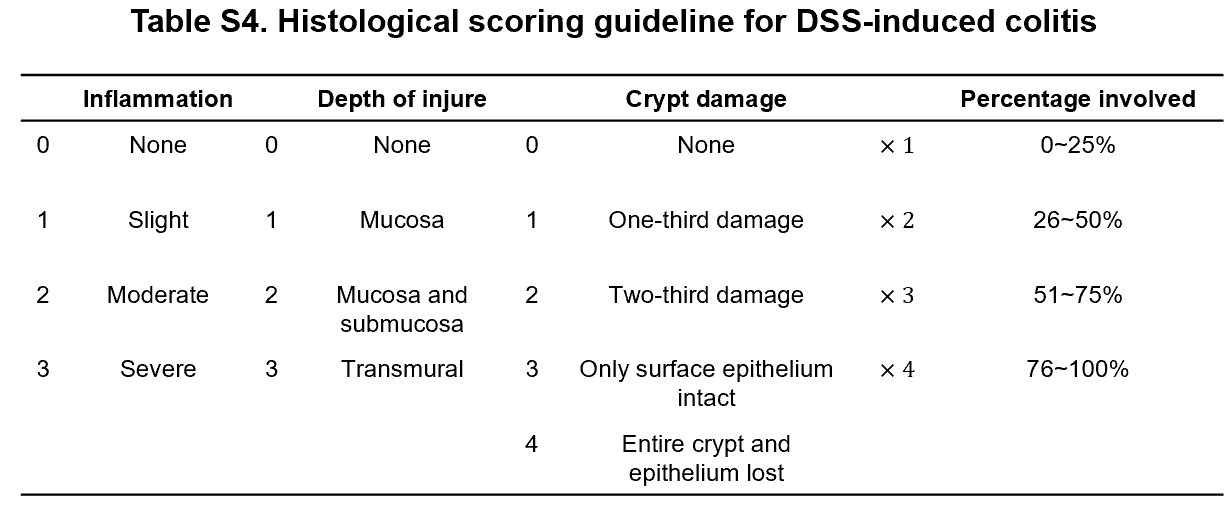


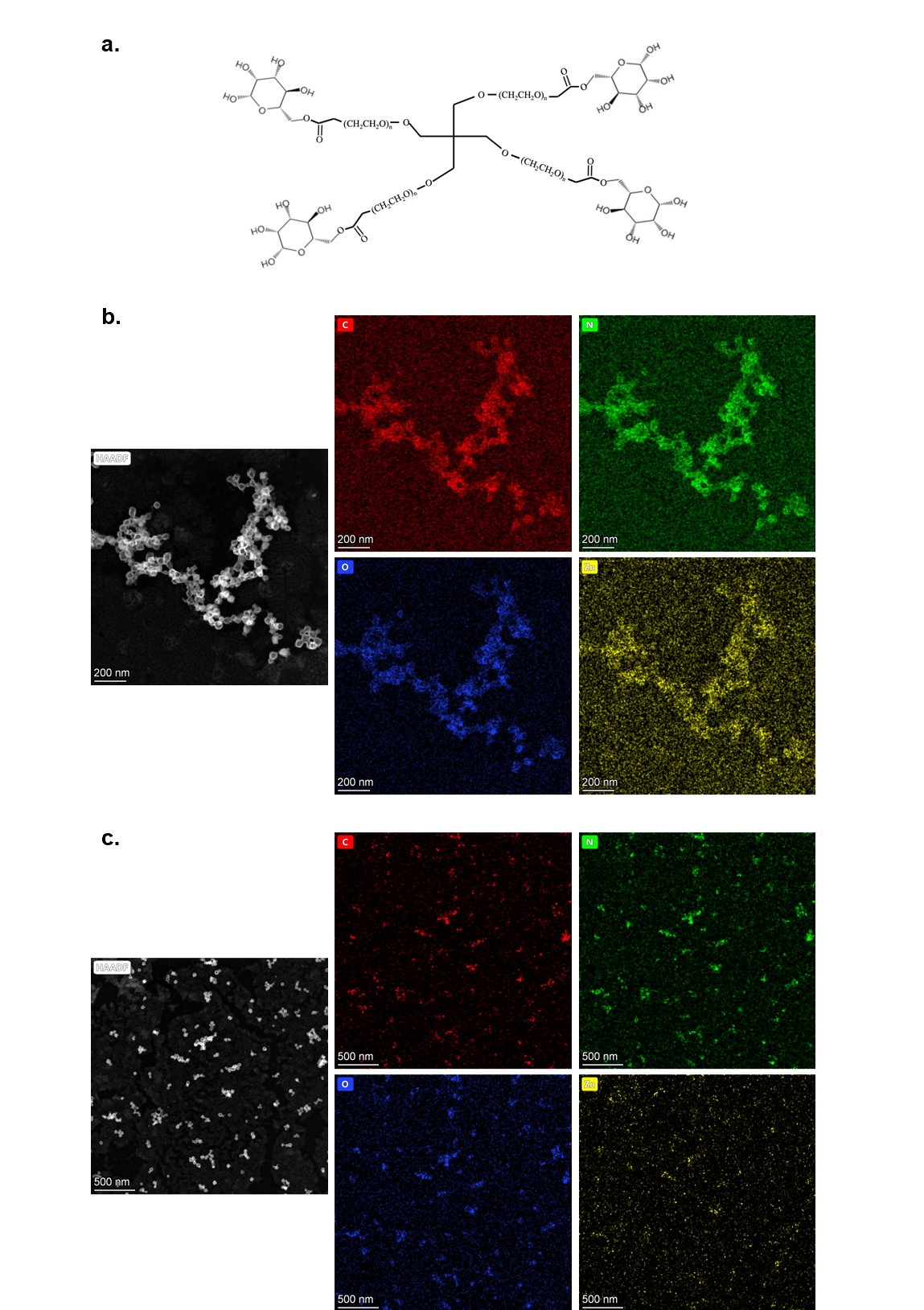


**Figure S1.** (a) Structural formula of 4-PEG-Man and element mapping of (b) PCNPs and (c) PCNPs@PEG-man. Scale bar: 200 nm


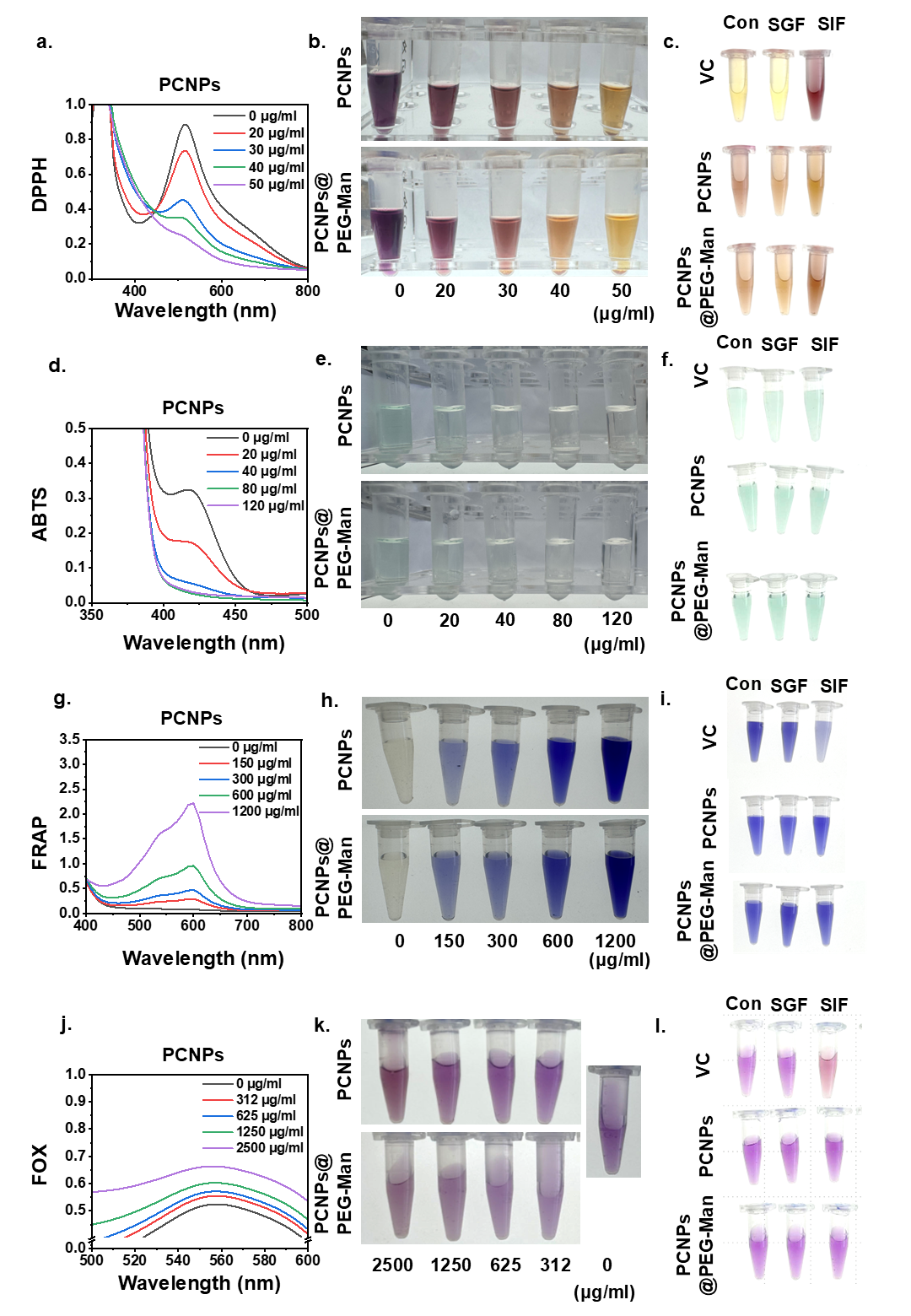


**Figure S2.** The antioxidant capacity colorimetric results of PCNPs and PCNPs@PEG-Man in various assays and in a simulated gastric and intestinal environment. (a-c) DPPH (absorbance at 517 nm), (d-e) ABTS (absorbance at 734 nm), (g-i) FRAP (absorbance at 593 nm), and (j-l) FOX (absorbance at 560 nm).


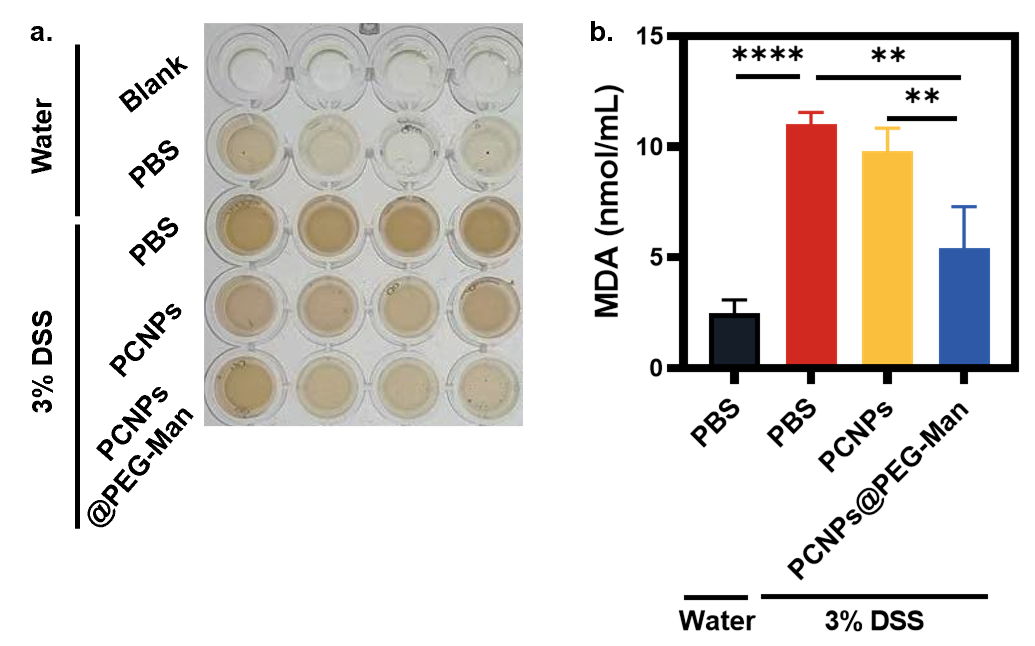


**Figure S3.** Measurement of malondialdehyde (MDA) levels in the plasma of DSS-induced colitis mice. (a)Color intensity of samples from each group in a 96-well plate assay. (b) Quantification of MDA levels in each group. Data are presented as mean ± SD (n = 4). Statistical analysis was performed using Student’s t-test. *P < 0.05, **P < 0.01, ***P < 0.001, ****P < 0.0001.

**
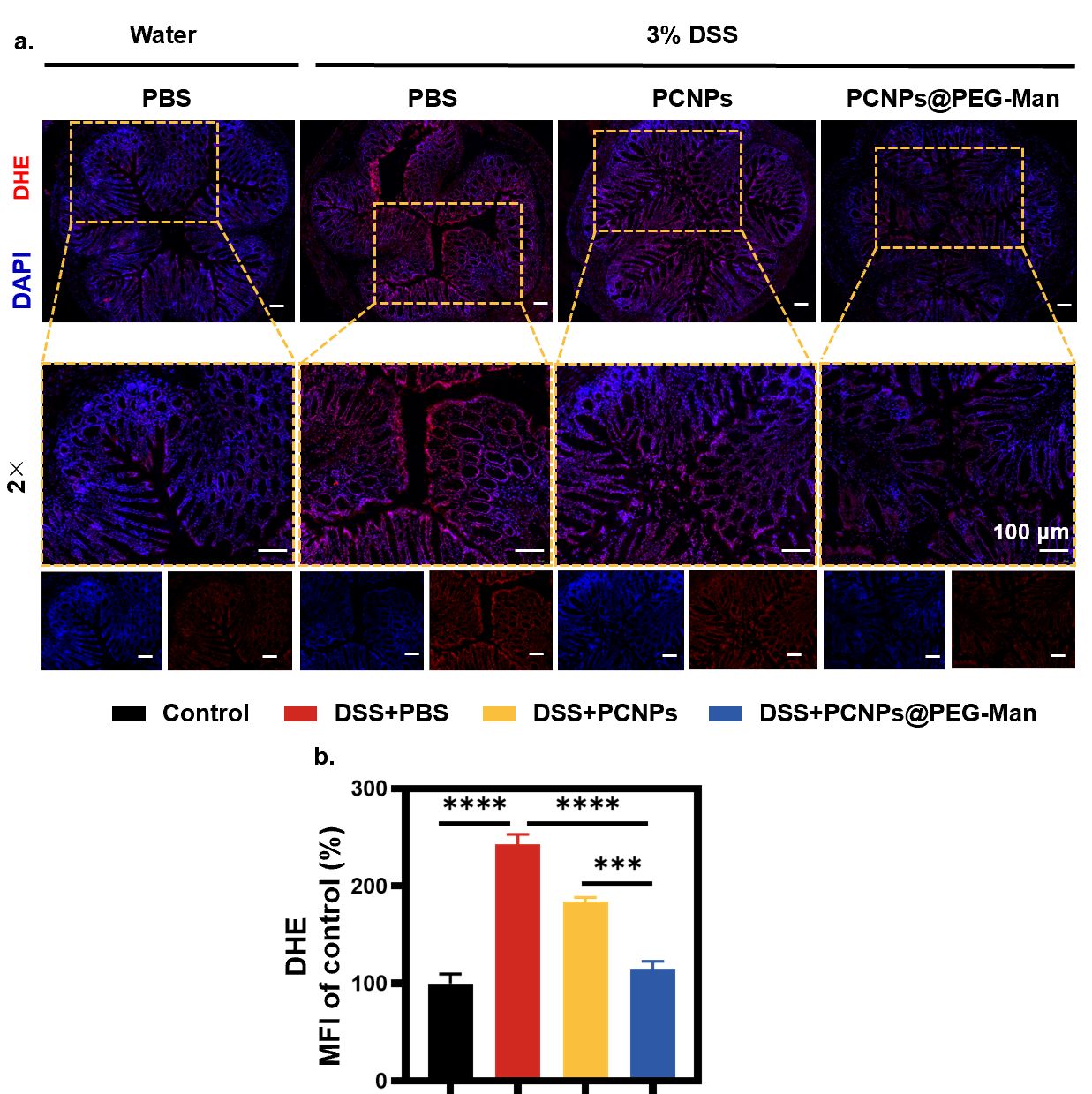
**

**Figure S4.** Detection of reactive oxygen species (ROS) in colonic tissues of mice with colitis. (a) Representative immunofluorescence (IF) staining images of dihydroethidium (DHE) and (b) relative quantitative analysis. Scale bar: 100 μm. Data are presented as mean ± SD (n = 3). Statistical significance was determined by Student’s t-test *P < 0.05, **P < 0.01, ***P < 0.001, ****P < 0.0001.


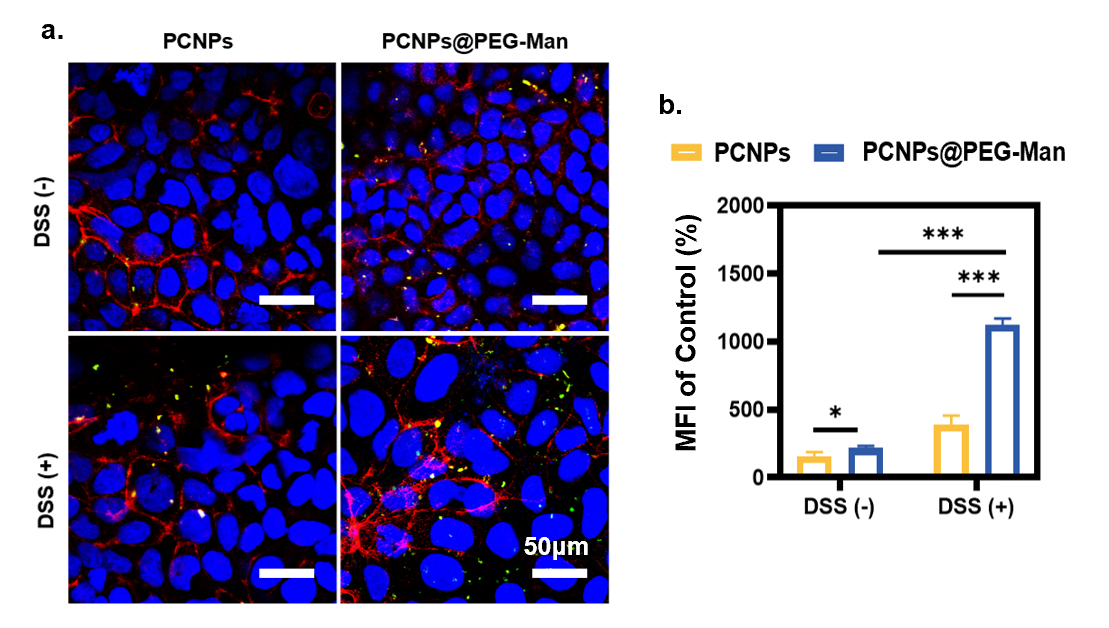


**Figure S5.** Cellular uptake behavior of FITC-labeled PCNPs and PCNPs@PEG-Man by RAW264.7 cells. (a) Confocal laser scanning microscopy (CLSM) images and (b) the corresponding semiquantitative fluorescence analysis. Data are mean ± SD (n = 3). Scale bar: 50μm. *P < 0.05, **P < 0.01, ***P < 0.001, ****P < 0.0001.


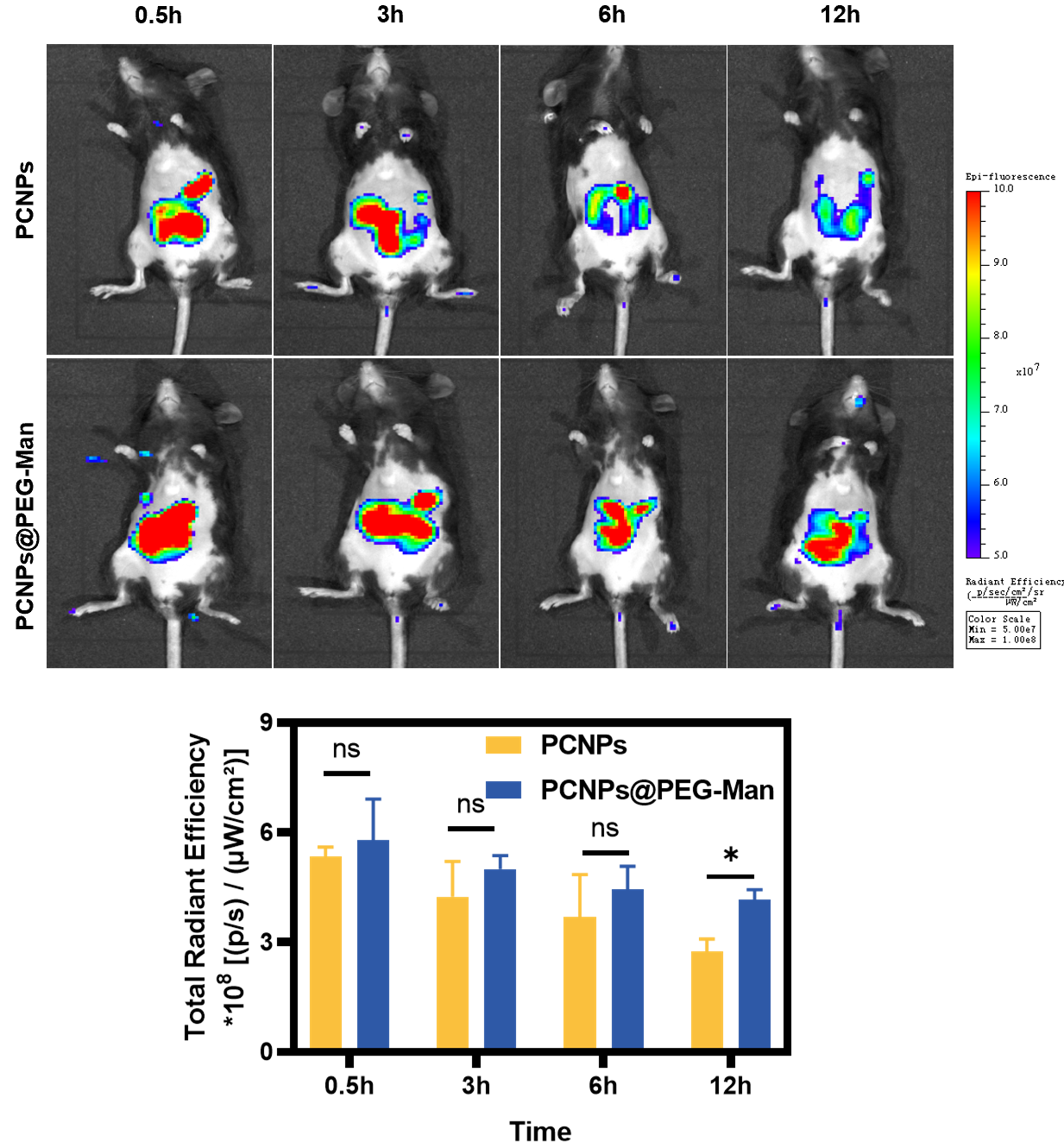


**Figure S6.** In vivo imaging of rhodamine-labeled PCNPs and PCNPs@PEG-Man in colitis mice at different time points. (a) Representative fluorescence images at 0.5 h, 3 h, 6 h, and 12 h post-administration. (b) Quantification of fluorescence intensity at each time point. Data are mean ± SD (n = 2). Statistical analysis was performed using Student’s t-test. *P < 0.05.
